# Multi-organ single-cell transcriptomic atlas identifies *QrIAA14* as a candidate negative regulator of adventitious root development in *Quercus robur*

**DOI:** 10.1371/journal.pgen.1012300

**Published:** 2026-09-01

**Authors:** Wenkai Hui, Jiayue Li, Hao Li, Hongyi Wu, Yi He, Shuangying Yang, Xiong Huang, Hanbo Yang, Gang Chen, Peng Zhu, Xiaohong Chen, Xingcui Xiao, Yu Zhong, Zhenfeng Xu, Fang He

**Affiliations:** 1 Forest Ecology and Conservation in the Upper Reaches of the Yangtze River Key Laboratory of Sichuan Province, College of Forestry, Sichuan Agricultural University, Chengdu, China; 2 Ecological Conservation, Restoration and Resource Utilization in Forest and Wetland Key Laboratory of Sichuan Province, Sichuan Academy of Forestry, Chengdu, China; University of Kentucky, UNITED STATES OF AMERICA

## Abstract

Plant organ development involves coordinated cell fate transitions across multiple tissues, yet the cellular programs underlying organ-specific differentiation in woody plants remain poorly understood, particularly the mechanisms limiting efficient root development during vegetative propagation of oak species. Here, we generated a comprehensive single-cell transcriptomic landscape of leaf, stem, and root tissues of *Quercus robur* to resolve developmental trajectories at cellular resolution. A total of 41,471 high-quality cells were classified into 30 distinct clusters, enabling the identification of major cell types and organ-specific transcriptional features across three vegetative organs. Pseudotime analyses exhibited the developmental programs related to guard cell differentiation in leaves, vascular formation in stems, and root tissue development. Additionally, combining scRNA-seq, bulk transcriptome profiling, and phytohormone investigations, we identified auxin signaling as an important regulator during adventitious root development process. Notably, *QrIAA14-1*, an *IAA14* homolog, was preferential enrichment in root hair, near-root hair cells and root cap along root developmental trajectories, which was further supported by RT-qPCR and *in situ* hybridization assays. Furthermore, the overexpression of *QrIAA14-1* significantly inhibited oak root elongation, resulting in around 64.46% reduction in adventitious root length compared with control plants, providing mechanistic insight into the limitations of root development in oak. Together, this study provides the first high-resolution single-cell atlas of cellular organization and developmental dynamics across oak vegetative organs and identifies the candidate regulator genes associated with root development, offering new insights into the regulatory mechanisms of woody plant root regeneration and clonal propagation.

## Author summary

Oaks are important forest trees with high ecological and economic value, but many oak species show poor efficiency in vegetative propagation because newly regenerated shoots often form roots inefficiently. This poor rooting ability limits the preservation and large-scale use of elite oak genotypes. In this study, we generated a single-cell transcriptomic atlas of leaf, stem, and root tissues in *Quercus robur*, revealing the cellular composition and developmental programs of major vegetative organs in oak. We further combined this single-cell resource with bulk RNA sequencing, phytohormone measurements, gene expression analyses, RNA *in situ* hybridization, and transgenic assays to investigate adventitious root development. Our results showed that auxin signaling is significantly associated with oak root regeneration. We identified *QrIAA14-1*, an auxin-related gene, as a candidate negative regulator of adventitious root development. Overexpression of *QrIAA14-1* reduced adventitious root elongation, suggesting that this gene may contribute to the limited rooting capacity of oak. These findings provide a useful resource for studying woody plant development and may help improve clonal propagation strategies in oaks.

## Introduction

Oaks (*Quercus* L.), the largest genus in the Fagaceae family with approximately 400–500 species, are keystone trees in temperate forests across the Northern Hemisphere [1]. Among them, *Quercus robur*, commonly known as English oak or pedunculate oak, is the most widespread, culturally iconic, and ecologically significant oak species in Europe. This species sustains highly complex ecosystems by providing habitats for hundreds of fungi and insect species and serving as a critical food source for birds and mammals, thereby contributing to overall ecosystem stability [2]. In addition, its wood, renowned for its hardness and aesthetic grain, had long been valued for shipbuilding, flooring, wine barrels, furniture and so on, while also carrying profound cultural significance throughout human history [3]. However, *Q. robur* is notoriously difficult to propagate vegetatively due to its poor rooting ability [4,5], which has severely hindered the rapid preservation and utilization of superior genotypes. Therefore, identifying key genetic regulators underlying the root formation is essential for developing effective strategies to enhance its clonal propagation in *Q. robur*, which could also offer new insights into root development in woody plants.

To date, auxin signaling has been widely recognized as a central regulator pathway governing root development, including adventitious root initiation, primordium formation and subsequent elongation [6]. At the core of this pathway, the perception of auxin by Transport Inhibitor Response 1/Auxin Signaling F-box (TIR1/AFB) receptors promoted the ubiquitin-mediated degradation of Auxin/Indole-3-Acetic Acid (AUX/IAA) transcriptional repressors, thereby releasing Auxin Response Factors (ARFs) to activate downstream transcriptional programs [7]. This regulatory module controls both the rapid early auxin responses, containing Small Auxin Up RNA genes (SAURs) and Gretchen Hagen 3 genes (GH3s), which modulated cell expansion and hormone homeostasis, as well as the transcriptional cascades derived cell-cycle re-entry, cell fate reprogramming and root primordium establishment. Some previous studies reported that AUX/IAA gene family acted as the core negative regulators in auxin signaling cascade [7–9]. The bZIP53-IAA4 module significantly suppressed adventitious root development in *Populus* [10], while the gain-of-function mutation of AUX/IAA stabilizes the protein, leading to persistent repression of ARF-mediated transcription and consequently blocking lateral root initiation [7,11,12]. However, the overexpression of rose *RhIAA25* promoted the adventitious root elongation in *Arabidopsis* but inhibited adventitious root formation [13]. Recently, the bamboo *PheIAA17* significantly enhanced lateral roots development by repressing *PheARF3-2* promoter activity [14]. These evidences indicated that AUX/IAA and ARF activities determined the plant root developments, and suggesting the functional diversification across plant species. Nevertheless, the cellular and molecular basis of auxin-mediated adventitious root formation remains poorly understood in oaks. Thus, exploring the potential IAA family members was an expected way to uncover critical regulatory nodes controlling root initiation and elongation in *Q. robur*.

Cells are the fundamental structure and functional units of life [15]. Research at the cellular level provides a refined perspective for understanding the developmental processes, yet cellular heterogeneity within the same tissue has posed major challenges in this field [16]. Recently, single-cell RNA (scRNA) sequencing technologies have offered powerful approaches to address this limitation by capturing transcriptomic information from individual cells while preserving their unique identities [17]. The annotation and classification of cell types based on specific marker genes are critical steps in single-cell analysis. To date, the major cell types of multi-organs, such as cotyledons, leaves, stems and roots, have been identified in *Arabidopsis* [18], rice [19], cotton [20], maize [21], poplar [22,23], *Eucalyptus* [24], and other plant species, leading to the discovery of key regulatory genes involved in phloem differentiation, xylem formation, root cap development, and stress responses [25,26]. However, most previous studies have focused on individual organs [21,27–29], which restricts to explore the organ-specific cell populations and identify tissue-specific regulatory programs. In recent years, the release of multiple *Quercus* genomes has provided valuable resources for molecular breeding [1,30–33]. However, the single-cell sequencing studies have not yet been reported in oaks, which has limited the key gene identification and functional exploration associated with adventitious root formation in *Q. robur*. To date, the scRNA sequencing via the 10x Genomics Chromium platform has emerged as the method of choice for plant cell atlas due to its high throughput and sensitivity [34]. However, application to woody species remains technically challenging due to lignified cell walls, high polyphenol content, and tissue heterogeneity, which can compromise protoplast viability and yield.

To address this, a high-resolution single-cell landscapes of *Q. robur* was established by isolating root, stem, and leaf from seedlings based on annotating specific marker genes and cell types, which enabled the reconstruction of developmental trajectories related to stomatal differentiation, xylem formation, and root elongation. Additionally, the comprehensive co-expression network governing adventitious root formation were established to identify the potential regulators with the integration of bulk RNA-seq and phytohormone profiles in four root development stages. Bioinformatic and functional validation assays demonstrated that IAA14 played important roles in regulating root formation in *Q. robur*. Our findings provided a single-cell level map of oak tissue development and offers valuable molecular targets for enhancing clonal propagation and advancing breeding strategies in woody plants.

## Results

### Construction of single-cell transcriptional landscape in *Q. robur*

**Fig 1:**
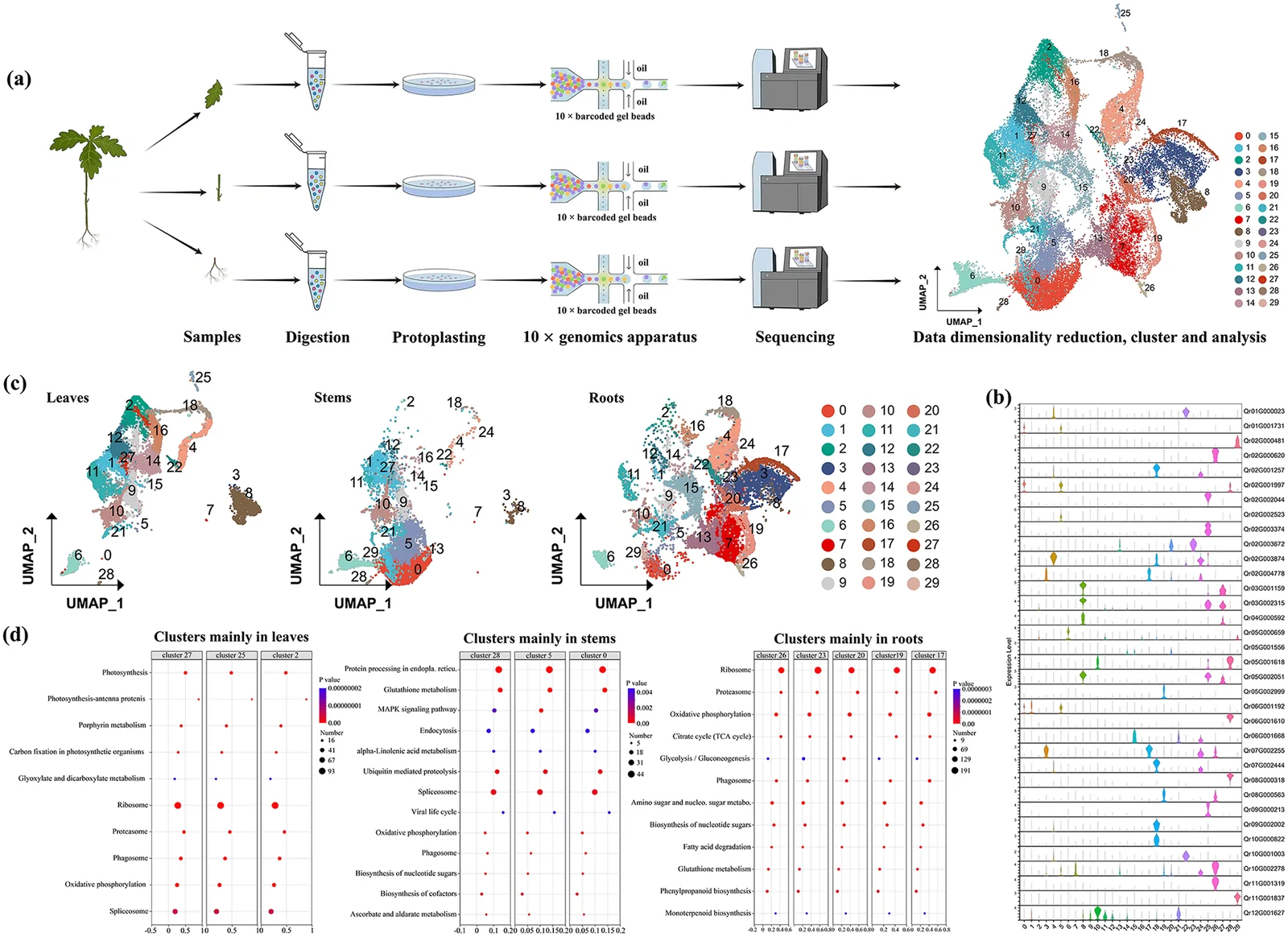
Cluster annotation and cell-type differences in single-cell RNA-seq analysis of three oak organs. a: Overview of the oak scRNA-seq and cluster annotation workflow, the protoplasts were isolated from leaf, stem, and root of tissue-cultured oak plantlets, each dot denotes a single cell. b: Violin plots showing expression of representative marker genes in each cell type, the colors denoted corresponding cell clusters. c: UMAP visualization of 30 putative clusters identified from leaves, stems and roots. Each dot denotes a single cell, colors indicate corresponding cell clusters. d: KEGG enrichment of the key clusters in each organ. The left y-axis represents the enrichment pathways, and the x-axis indicates the percentages of DEGs belonging to the corresponding pathway. The sizes of bubble represent the number of DEGs in the corresponding pathway, and the colors of the bubble represent the *P* value of the corresponding pathway.

To generate a comprehensive single-cell transcriptomic dataset for oak, the leaf, stem, and root tissues were carefully dissected (Fig 1a) and enzymatically digested to isolate free protoplasts, which were subsequently used for single-cell library construction on the 10x Genomics platform. Following stringent quality filtering at both the cell and gene levels, a total of 52,559 single cells were retained from an initial sequencing output of 494.12 Gb raw data (S1 Table), and the median number of detected genes per cell varied between 597 and 2,912. Valid barcode assignment rates exceeded 96.5% in all libraries, with GC contents ranging from 38.0% to 40.5%. After dimensionality reduction based on gene expression patterns and clustering with Seurat’s Find Clusters function (resolution = 0.8) (Fig 1a and 1b), a total of 41,471 high-quality single cells were classified into 30 distinct clusters (cluster 0 to cluster 29), with cluster 0 containing the largest number of cells (5,943 cells) and cluster 29 the fewest (77 cells) (S2 Table). Collectively, these quality metrics demonstrate that a robust and high-quality single-cell transcriptomic resource was established for *Q. robur*, providing a reliable foundation for downstream cell-type identification and developmental trajectory analyses.

Further analysis revealed the significant differences in the distribution of cell clusters among three organs (S1 Fig and S2 and S3 Tables). The leaf-derived cells were predominantly distributed in multiple clusters (Fig 1c), with cluster 2 (2,653/2,704), cluster 25 (123/123), and cluster 27 (103/104) consisting of more than 98% of cells (S2 Table), indicating that these clusters represented leaf-dominant cell populations. KEGG enrichment of the three clusters revealed the significant involvement in photosynthesis and protein biosynthesis pathways (Fig 1d), suggesting that these clusters may associated with the cellular basis for nutrient biosynthesis and accumulation in *Q. robur* leaves. Additionally, the stem cells were mainly distributed in eight clusters (cluster 0, 1, 5, 6, 9, 10, 21, and 28), with the highest proportions observed in cluster 0 (94.6%), cluster 5 (92.1%), and cluster 28 (67.4%) (S2 Table). KEGG enrichment indicated that these stem-related clusters were mainly related to protein processing, signal transduction, and oxidative phosphorylation (Fig 1d), highlighting their potential roles in nutrient processing and transport. Notably, root-derived cells exhibited the broadest distribution among the clusters (S2 Table), which were detected in all clusters except cluster 25, cluster 27, and cluster 28. Clusters 17, 19, 20, 23, and 26 were exclusively composed of root cells, and mainly associated with glycolysis/gluconeogenesis, oxidative phosphorylation, citrate cycle, phenylpropanoid biosynthesis, and monoterpenoid biosynthesis pathways (Fig 1d), suggesting that it may played central roles in energy production, metabolic regulation, and the biosynthesis of structural components required for root differentiation and development in root tissues of *Q. robur*.

### Cell types identification and differentiation trajectory of leaf tissue

**Fig 2:**
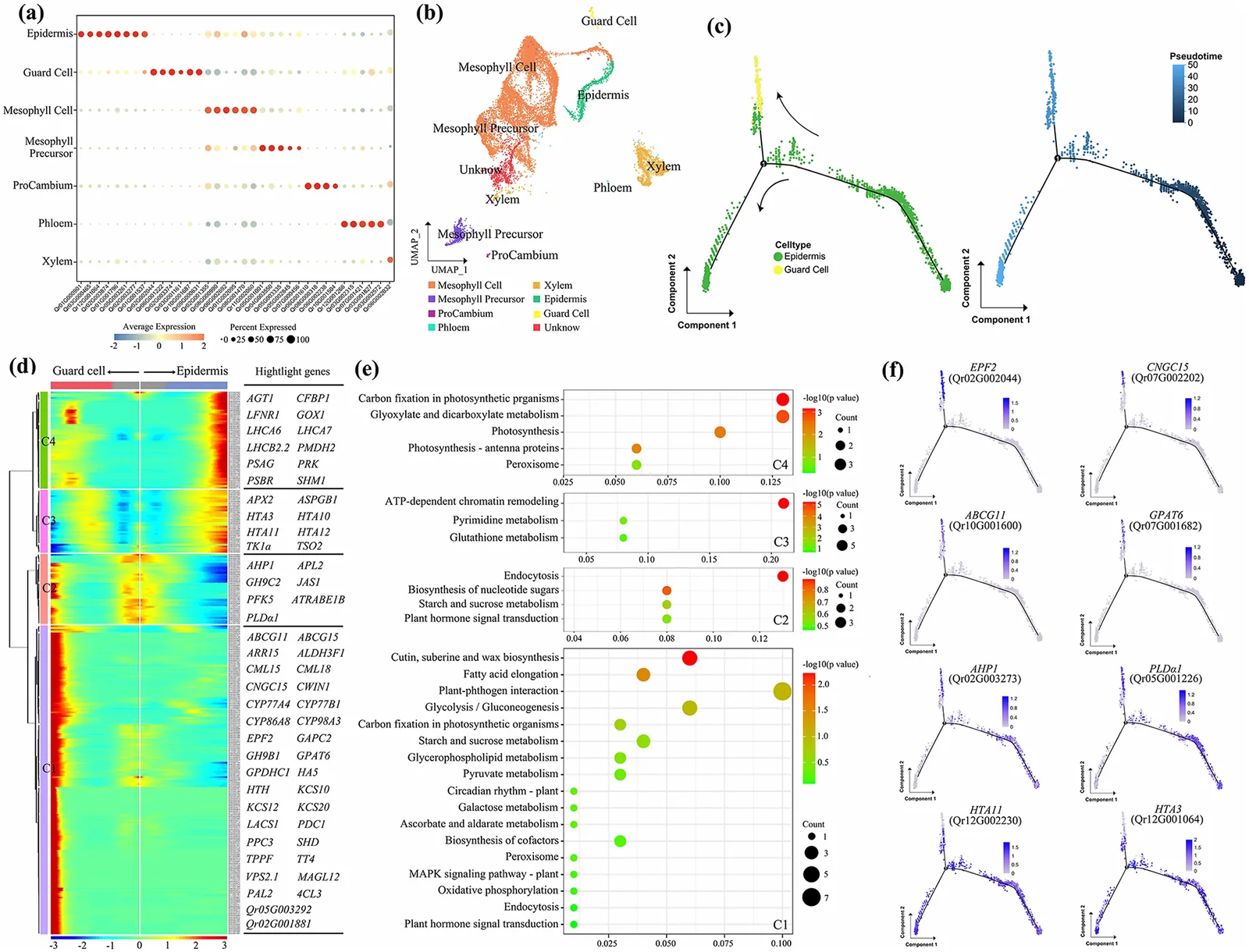
Pseudo-time trajectory and regulation pathways of leaf cells based on single-cell RNA-seq. a: The expression patterns of marker genes to identify the cell types in leaves. Dot size indicates the proportion of cells expressing each gene, and color intensity represents the average expression level. b: UMAP visualization of cell types identified from oak leaves, including guard cell, mesophyll cell, mesophyll precursor, epidermis, xylem, and phloem cells. c: Pseudo-time trajectory analysis of epidermal and guard cell lineages. Cells are colored by cell type and pseudo-time progression, illustrating a continuous differentiation path from epidermal cells to guard cells. Arrows indicate the inferred direction of cell state transitions. d: Heatmap showing dynamic gene expression patterns along the epidermis-guard cell differentiation trajectory, and representative key DEGs for each module are highlighted. e: KEGG enrichment of the genes based on the four gene clusters in heatmap. Dot size represents the number of genes enriched in each pathway, and color indicates the statistical significance. f: The expression patterns of the key genes related to epidermis and guard cell development along the inferred trajectory, *EPF2* (*EPIDERMAL PATTERNING FACTOR 2*), *CNGC 15* (*Cyclic Nucleotide‑Gated Ion Channel 15*), *ABCG11* (*ATP‑Binding Cassette Transporter G 11*), and *GPAT6* (*Glycerol‑3‑Phosphate Acyltransferase 6*), *HTA* (*Histone H2A), AHP* (*Arabidopsis Histidine Phosphotransfer Protein*), *PLDα1* (*Phospholipase D alpha 1*).

Based on the marker genes collected in this study, seven major cell types were identified from the clusters related to oak leaves (S4 Table), including epidermis, guard cells, mesophyll cells, mesophyll precursors, xylem, phloem, and procambium (Fig 2a). The UMAP visualization further showed the strong cell-type-specific expression patterns, with epidermal and guard cell clusters displaying distinct transcriptional signatures compared with mesophyll-derived lineages (Fig 2b). Given the pivotal role of guard cells in stomatal movement and plant stress responsiveness, this study further focused on exploring key genes and the developmental trajectories of epidermal and guard cell lineages. The trajectory analysis reconstructed a continuous differentiation process from epidermal cells toward guard cells, supported by a coherent pseudotime gradient (Fig 2c). Along this trajectory, epidermal cells were predominantly distributed at early pseudotime stages, whereas guard cells accumulated toward later stages, indicating progressive cellular specialization during stomatal development.

Furthermore, all the genes involved in epidermal and guard cells divided into four distinct transcriptional clusters with stage-specific activation patterns (Fig 2d). Among these, the C1 cluster was mainly significant upregulated in guard cells, C2 was detected in both guard cell and epidermis, while C3 and C4 was predominantly expressed in epidermis cells, reflecting cell-type-specific transcriptional regulation during leaf epidermal differentiation. KEGG pathway analysis revealed that the genes in C1 clusters were significantly enriched in energy metabolism, photosynthetic carbon assimilation, and cuticular formation pathways, including glycolysis/gluconeogenesis, carbon fixation in photosynthetic organisms, and cutin, suberine and wax biosynthesis (Fig 2e and S5 Table), which aligns closely with their physiological roles in stomatal movement, osmoregulation, and the construction of hydrophobic protective layers. Consistently, four highlight genes in C1 dataset exhibited characteristic guard cell-specific expression dynamics along the pseudotime trajectory (Fig 2f), including Epidermal Patterning Factor (*EPF2*), Cyclic Nucleotide-Gated Channel 15 (*CNGC15*), ATP-Binding Cassette G11 (*ABCG11*), and Glycerol-3-Phosphate Acyltransferase 6 (*GPAT6*), which have been implicated in guard cell formation, stomatal closure, ion transport, and cuticular lipid biosynthesis, reinforcing the functional identity of the guard cell differentiation program captured at the single-cell level.

Additionally, the genes in the C2 cluster displayed sustained transcriptional activity across both epidermal and guard cells, with enhanced expression in guard cells. These genes were enriched in pathways associated with endocytosis, biosynthesis of nucleotide sugars, and phytohormone signal transduction, reflecting coordinated regulation of membrane trafficking, cell wall precursor supply, and signal integration during epidermal-to-guard cell transition (Fig 2f). Moreover, epidermal cell-enriched gene clusters, C3 and C4, were significantly associated with pathways related to photosynthesis and antenna protein assembly, chromatin remodeling, carbohydrate metabolism, and lipid biosynthesis (Fig 2d and 2e). For example, two genes, HISTONE H2A 3 (*HTA3*) and HISTONE H2A 11 (*HTA11*) annotated with histone H2A protein family, exhibited predominately epidermis-enriched expression patterns (Fig 2f). These results suggest that epidermal cells might maintain a highly controlled chromatin state supporting environmental perception and developmental plasticity prior to stomatal lineage. Collectively, these findings defined the cellular composition of oak leaves at single-cell resolution and revealed a coordinated, stage-dependent transcriptional program underlying epidermal differentiation and guard cell development.

### Cell types identification and differentiation trajectory of stem tissue

**Fig 3:**
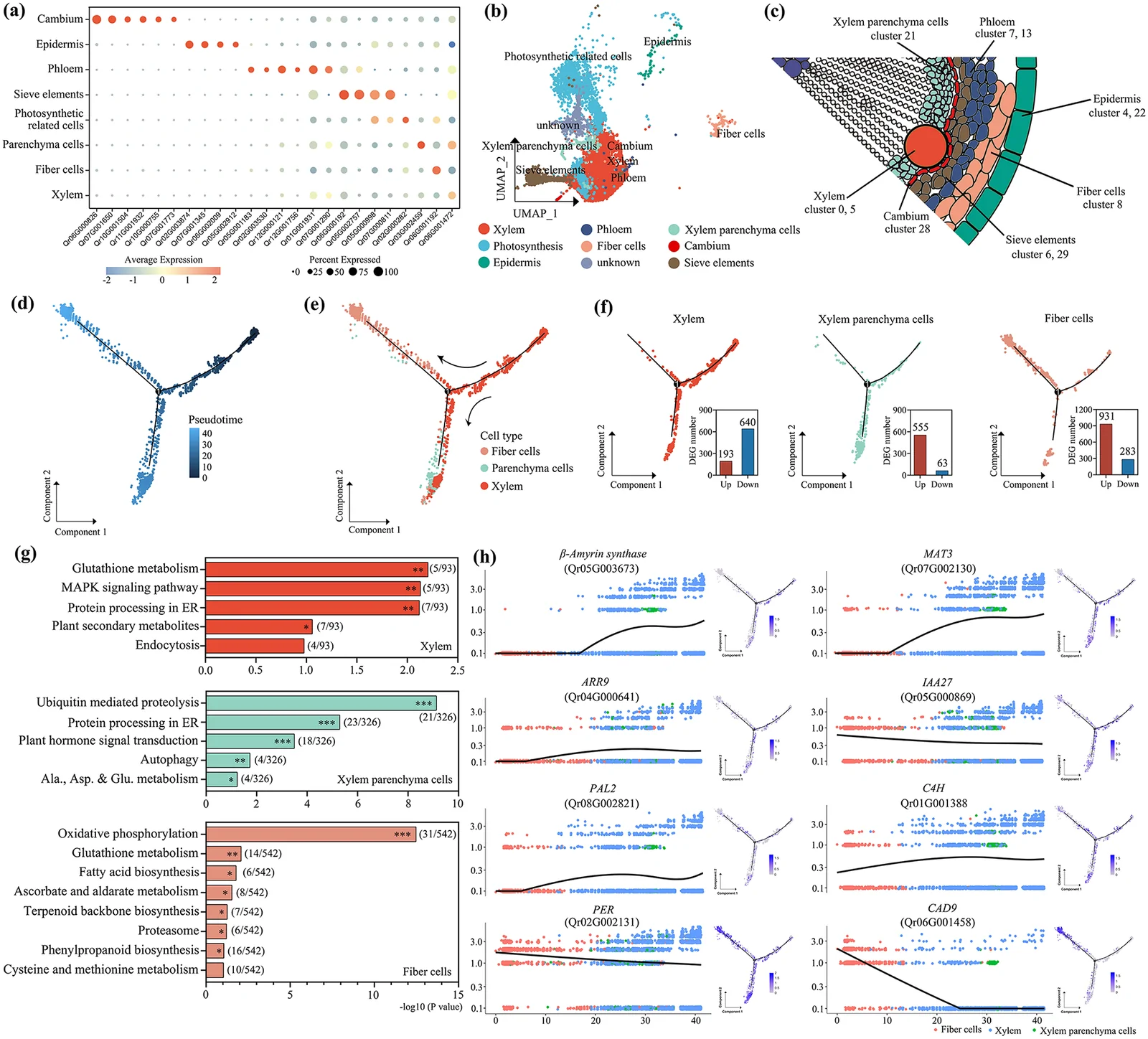
Pseudo-time trajectory and regulation pathways of stem cells based on single-cell RNA-seq. a: The expression patterns of marker genes across major stem cell types. Dot size indicates the proportion of cells expressing each gene, and color intensity represents the average expression level. b: UMAP visualization of cell types identified in oak stems, each dot denotes a single cell, colors indicate corresponding cell clusters. c: Schematic visualization of cell structure and cluster distribution in stem. d-f: Pseudo-time trajectory of fiber, xylem and xylem parenchyma cells, each dot indicates a single cell, the black arrow indicates the directions of differentiation, and the bar charts were the DEGs identified in each cell type. g: KEGG enrichment of branch-specific DEGs for xylem, xylem parenchyma cells, and fiber cells, Bar length represents enrichment significance, the * represents significant enrichment (*P* < 0.05), the ** represents very significant enrichment (*P* < 0.01), and the *** represents extremely significant enrichment (*P* < 0.001). h: Expression dynamics of key genes related to fiber, xylem and xylem parenchyma cell development along the differentiation trajectories, the horizontal axis represents pseudo-time, the vertical axis represents relative gene expression abundance. Each dot indicates a single cell, and red represents fiber cells, blue represents xylem cells, and green represents xylem parenchyma cells, *MAT* (*METHIONINE ADENOSYLTRANSFERASE*), *ARR* (*ARABIDOPSIS RESPONSE REGULATOR*), *IAA* (*AUXIN/INDOLE-3-ACETIC ACID*), *PAL* (*PHENYLALANINE AMMONIA-LYASE*), *C4H* (*CINNAMATE 4-HYDROXYLASE*), *PER* (*PEROXIDASE*), *CAD9* (*CINNAMYL ALCOHOL DEHYDROGENASE*).

To characterize vascular differentiation dynamics in oak stems, single-cell transcriptomic profiling was performed to resolve multiple vascular- and epidermis-associated cell populations, including cambium, xylem, xylem parenchyma cells, fiber cells, phloem, sieve elements, epidermis, and photosynthesis-related cells (Fig 3a and 3b). These cell populations displayed clear transcriptional separation, reflecting the complex cellular composition of stems in woody plants.

Additionally, UMAP analysis further revealed that cambial cells were transcriptionally positioned adjacent to multiple vascular cell types, consistent with their close developmental association with differentiated vascular tissues during secondary growth (Fig 3b and 3c). To specifically investigate differentiation programs within mature vascular tissues, the pseudotime analysis was conduct using the clusters of xylem, xylem parenchyma, and fiber cells (Fig 3d). The reconstruction trajectory was captured a progressive transcriptional divergence among xylem, xylem parenchyma cells, and fiber cells, reflecting the distinct differentiation programs within vascular tissues of oak stems (Fig 3d–3f). To explore transcriptional reprogramming accompanying this process, DEGs significantly up-regulated in each cell type were identified and subjected to KEGG enrichment analysis (Fig 3e and 3f). Genes enriched along the xylem branch were primarily associated with plant secondary metabolism and signaling-related pathways, consistent with the specialized roles of xylem in transport and structural support. The xylem parenchyma cells showed enrichment of genes related to protein turnover, phytohormone signal transduction, and primary metabolic processes (S6 Table). However, the fiber cell branch exhibited strong enrichment of genes associated with energy metabolism, secondary metabolism, and cell wall-related biosynthetic pathways (Fig 3g). Consistent with these functional distinctions, the DEGs implicated in vascular development of the three cell types displayed branch-specific and dynamic expression patterns along the differentiation trajectories (Figs 3h and S2), including *β*-amyrin synthase, Methionine Adenosyl-transferase 3 (*MAT3*), Response Regulator 9 (*ARR9*), *IAA27*, Phenylalanine Ammonia-Lyase 2 (*PAL2*), Cinnamate 4-Hydroxylase (*C4H*), Peroxidase (*PER*), and Cinnamyl Alcohol Dehydrogenase 9 (*CAD9*) genes.

Together, these results delineate the transcriptional landscape and internal differentiation continuum of vascular tissues in oak stems at single-cell resolution, highlighting coordinated yet specialized programs underlying functional diversification of woody vascular cells.

### Cell types identification and differentiation trajectory of root tissue

**Fig 4:**
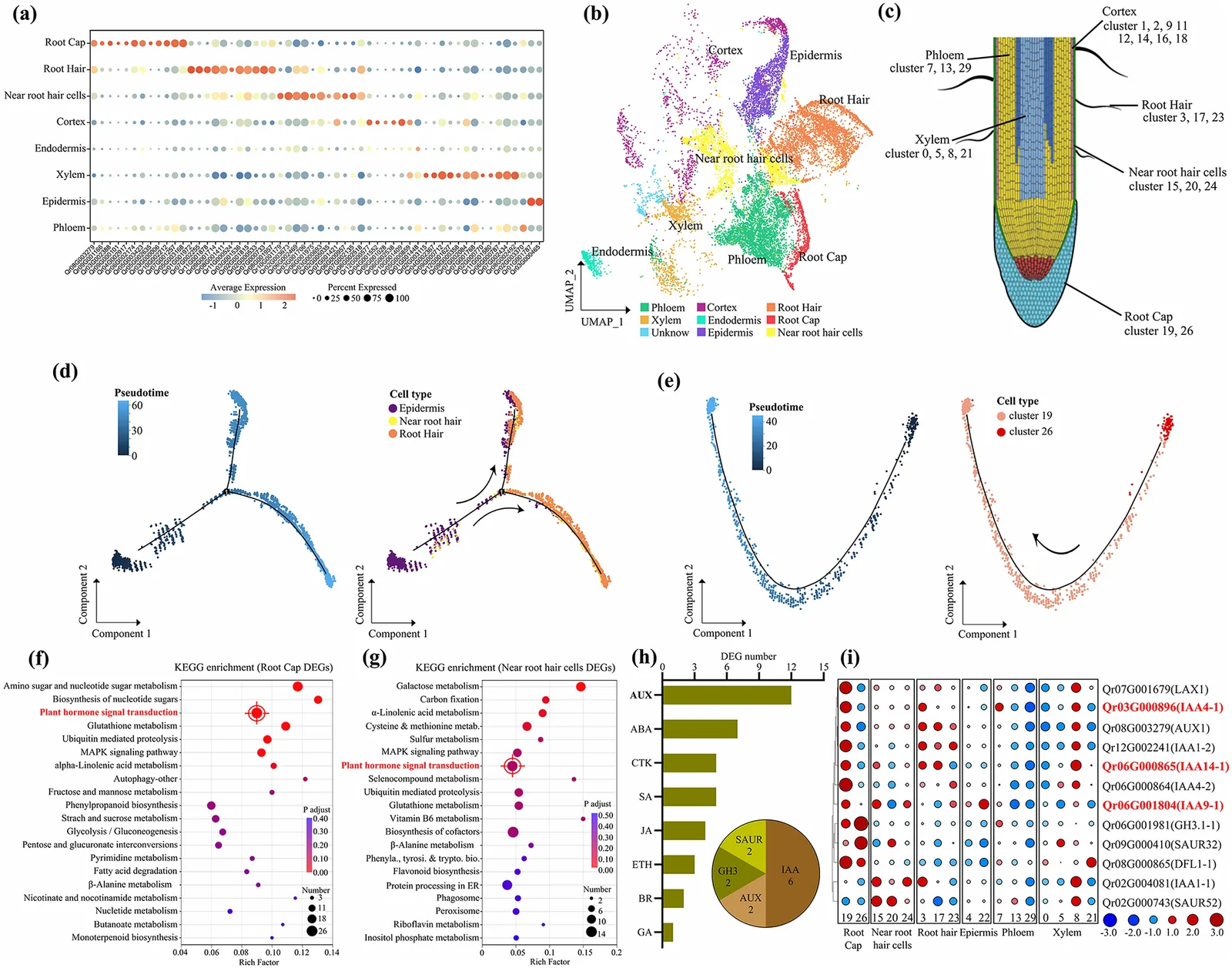
Single-cell resolution analysis reveals cellular organization and hormone-regulated differentiation of oak roots. a: The expression patterns of marker genes to identify the cell types in roots. Dot size indicates the proportion of cells expressing each gene, and color intensity represents the average expression level. b: UMAP visualization of cell types identified in oak roots. c: Schematic visualization of cell structure and clusters distribution in roots. d: Pseudo-time trajectory of epidermis, root hair and near root hair cells, each dot indicates a single cell, the black arrow indicates the start of the trajectory. e: Pseudo-time analysis of root cap cells identified from cluster 19 and 26. f-g: KEGG enrichment of the DEGs in root cap and near root hair cells. h: Distribution of DEGs significantly enriched in phytohormone signal transduction pathway. i: Expression heatmap of the DEGs in auxin signaling pathway identified in KEGG enrichment analysis.

Based on the transcriptional signatures of marker genes, root cell clusters were classified into epidermis, root hair cells, near root hair cells, cortex, endodermis, xylem, phloem, and root cap (Fig 4a and 4b). These cell populations formed well-separated clusters in UMAP space, reflecting the highly organized cellular architecture of the root system.

Mapping transcriptional clusters onto root anatomy revealed a clear correspondence between single-cell-defined populations and tissue layers (Fig 4c). Notably, epidermis, near root hair, and root hair cells constituted a connected transcriptional domain, suggesting a continuous differentiation relationship among these cell types (Fig 4b). This observation prompted further investigation into epidermal lineage progression during root hair development. Pseudotime trajectory analysis reconstructed a developmental continuum originating from epidermis cells, transitioning through near root hair cells to terminate in mature root hair cells (Fig 4d and 4e). Along this trajectory, cells displayed gradual transcriptional states changes rather than abrupt transitions, indicating progressive specialization during root hair formation. Given the essential role of the root tip in root growth, differentiation, and nutrient absorption, two clusters annotated as root cap cells, cluster 19 and cluster 26, were collected to performed pseudotime trajectory analysis. The results revealed a directional progression from cluster 26 to cluster 19, accompanied by marked differences in gene expression patterns, suggesting dynamic transcriptional regulation during root cap development (Fig 4e).

To explore molecular programs underlying oak root development, we focused on root cap and near root hair cell populations in this study, which were closely associated with root tip function and epidermal differentiation, respectively. KEGG enrichment analysis found that the DEGs from both cell types were significantly enriched in phytohormone signal transduction pathway, reflecting the central role of hormonal regulation in root development (Fig 4f and 4g). Among these hormone-related genes, auxin signaling components represented the largest proportion, with 12 auxin-associated genes identified, followed by ABA (7 genes), cytokinin (5 genes), and salicylic acid (5 genes) signaling pathways (Fig 4h). Furthermore, AUX/IAA family members constituted the majority of auxin-related DEGs. Three *IAA* genes, Qr03G000896 (*IAA4-1*), Qr06G000865 (*IAA14-1*), and Qr06G001804 (*IAA9-1*), were significantly upregulated in root cap, near root hair and root hair cells, suggesting their potential central roles in regulating oak root development (Fig 4i).

### Root development observation and bulk RNA-seq analysis

**Fig 5:**
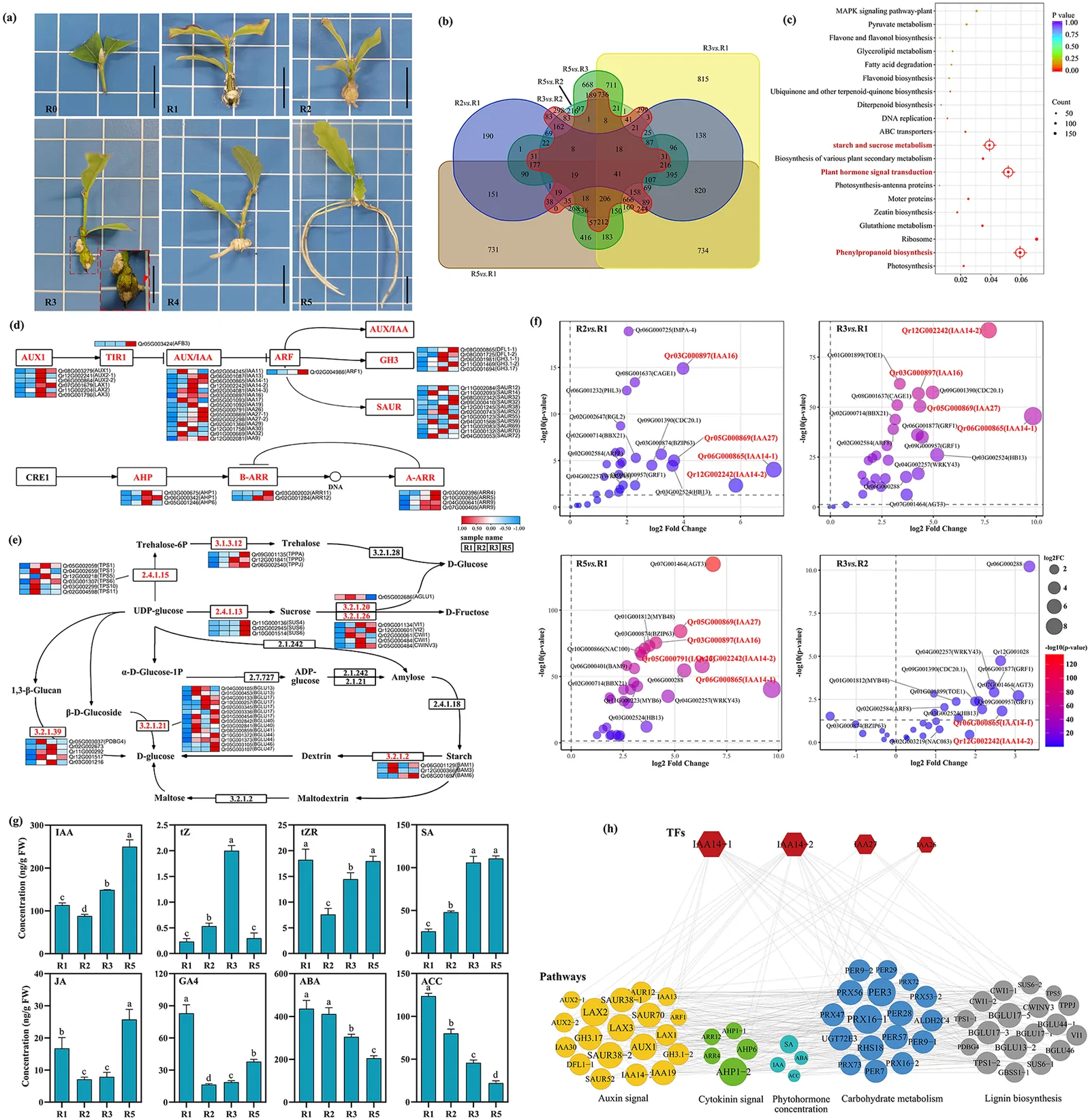
Identification of candidate genes associated with adventitious root development based on RNA-seq. a: Morphological observation of the adventitious root growth in oak tissue culture seedlings, the red arrow highlights the newly emerged adventitious root tip in R3 stage, the callus and/or adventitious root tissues were collected from the basal rooting zone of plantlets at the R1, R2, R3, and R5 stages for bulk RNA-seq, RT-qPCR, and phytohormone quantification. b: Venn diagram of DEGs in six comparisons, including R5*vs.*R1, R2*vs.*R1, R3*vs.*R2, R5*vs.*R2, R5*vs.*R3, R3*vs.*R1. c: KEGG enrichment of all the non-redundant DEGs detected from six comparisons. d: Heatmap of DEGs enriched in auxin and cytokinin signaling transduction pathways. e: DEGs enriched in starch and sucrose metabolism pathways. f: The key transcriptional regulator identification during oak root growth, the bubble size represents the log2(fold change), and the red font highlights the IAA genes in TOP15 transcriptional regulators in each comparison. g: the phytohormones investigation during oak root growth, IAA: indole-3-acetic acid, tZ: trans-zeatin, tZR: trans-zeatin riboside, SA: salicylic acid, JA: Jasmonic acid, GA4: gibberellin A4, ABA: abscisic acid, ACC: 1-aminocyclopropane-1-carboxylic acid and a direct precursor of ethylene, FW: fresh weight, the different low-case represents the significant differences, *P* < 0.05. h: Co-expression network of four *QrIAA* candidates and key transcriptional regulators in significantly enriched pathways. The bubble and font sizes were the log2(fold change) of R1*vs.* R3 for each DEGs.

The adventitious root development of *Q. robur* plantlets was divided into six distinct stages during tissue culture propagation (Fig 5a). At the R0 stage, explants consisted of 2–3 cm stem segments with axillary buds and partial leaves. After approximately one week, the callus tissue gradually emerged through the epidermis, marking the initiation of adventitious root induction (R1 stage). By 10–12 days after culture, callus tissues obviously proliferated accompanied by adventitious root primordium initiation (R2 stage). Around two weeks after inoculation, the root differentiation become evident and adventitious root tips appeared (R3 stage), followed by progressive adventitious root elongation after three weeks (R4 stage). By approximately five weeks (R5 stage), a well-developed root system was formed (R5), enabling the successful transplantation of plantlets. Based on these observations, samples from the R1, R2, R3, and R5 stages were selected for transcriptome sequencing. In total, 89.91 Gb of sequencing data were generated (S7 Table). After quality filtering, 6.33-8.70 Gb of clean bases were obtained in each sample with GC contents ranging from 44.15% to 45.77% and Q30 values consistently exceeding 95%. Mapping rate to the reference genome ranged from 78.97% to 91.18% (S8 Table), confirming the establishment of a high-quality transcriptome dataset for adventitious root development in *Q. robur*.

To comprehensively investigate the genetic regulation of root development in *Q. robur*, a total of 12,175 non-redundant DEGs were identified across six comparisons (Fig 5b). KEGG analysis revealed that these DEGs were significantly enriched in phytohormone signal transduction, starch and sucrose metabolism, and phenylpropanoid biosynthesis pathways (Fig 5c), suggesting adventitious root formation in *Q. robur* is primarily governed by hormone signaling, energy metabolism, and lignin biosynthesis. Phytohormones play pivotal roles in regulating growth and development. In this study, 91 DEGs were significantly associated with phytohormone signaling transduction (S9 Table), 41 of which (43.15%) were related to auxin signaling (Fig 5d). These DEGs were annotated with the entire auxin signaling cascade, highlighting auxin as a key regulatory factor in adventitious root development of *Q. robur*. Notably, DEGs encoding AUX/IAA and SAUR were most abundant and markedly up-regulated at the R2, R3, and R5 stages, suggesting their crucial roles in the initiation, differentiation, and maturation of root development in *Q. robur*. In addition, several DEGs associated with the cytokinin signaling pathway (Fig 5d), including members of the Histidine Kinase (*AHK*), Histidine Phosphotransfer Protein (*AHP*), Type-B Response Regulator (*B-ARR*), and Type-A Response Regulator (*A-ARR*) families, exhibited significant up-regulation at the R3 and R5 stages, indicating that cytokinin may contribute to the root developmental and elongation in *Q. robur*. Moreover, carbohydrates also exhibited strong transcriptional activation during rooting. A total of 64 DEGs were significantly enriched in starch and sucrose metabolism pathway, related to the biosynthesis of starch, sucrose, fructose, glucose, maltose, and trehalose (Fig 5e). All these DEGs exhibited significantly up-regulation in root differentiation stages, with the most enriched in *β*-glucosidase family annotated with the conversion of *β*-D-glucosides into D-glucose. These findings suggest that carbohydrates may play the critical roles in energy supplement and adventitious root development in *Q. robur*.

### Phytohormone investigation during root development

To further elucidate the phytohormonal regulation of rooting, endogenous phytohormone levels were quantified using the HPLC-ESI-MS platform (Fig 5g), and the results showed the significant changes during the adventitious root development in *Q. robur*. Indole-3-acetic acid (IAA) exhibited a significant increase during root differentiation process, especially in the R5 stage reaching approximately 2.2-fold higher than those at R1, which was consistent with the RNA-seq analysis and supporting the pivotal role of IAA in adventitious root initiation and development in *Q. robur*. Additionally, the content of trans-zeatin (tZ), a kind of cytokinin compounds, were increased in root differentiation, reaching 4–5 times higher at R3 stage compared with R1 stage. However, trans-zeatin riboside (tZR) displayed a down-regulation pattern, with a marked decline during the early stages of root induction. These results suggest that tZ may act as the predominant cytokinin driving adventitious root development in *Q. robur*. Conversely, the levels of abscisic acid (ABA), gibberellin acid 4 (GA_4_), and the ethylene precursor 1-aminocyclopropane-1-carboxylic acid (ACC) were showed a significantly down-regulation during the root development process, implying that these hormones may play limited roles in root differentiation of *Q. robur*.

### *QrIAA14-1* overexpression impairs adventitious root elongation

Transcriptional regulators (TRs) and transcription factors (TFs) play important roles in regulating plant growth and development. In this study, the TR and TF candidate datasets were identified in six comparison groups of bulk RNA-seq dataset, and the TOP30 results revealed that these DEGs were significantly associated with auxin signaling transduction (Fig 5f), especially Qr06G000865 (*QrIAA14-1*), Qr12G002242 *(QrIAA14-2*), Qr03G000725 (*QrIAA16*), Qr05G000791 (*QrIAA26*), and Qr05G000869 (*QrIAA27*) consistently exhibiting marked differential expression in all comparisons, suggesting their potential involvement in root development of *Q. robur*. Further co-expression network analysis demonstrated that *QrIAA14-1*, *QrIAA14-2*, *QrIAA26*, and *QrIAA27* showed significant associations with the enriched pathways in adventitious root development (Fig 5h), particularly auxin signaling, carbohydrate metabolism, and lignin biosynthesis, whereas *QrIAA16* did not detect significant co-expression patterns. Collectively, these findings highlighted *QrIAA14-1*, *QrIAA14-2*, *QrIAA26*, and *QrIAA27* were identified as candidate AUX/IAA regulators potentially involved in adventitious root development in *Q. robur*.

Considering the typical role of AUX/IAA repressors in modulating ARF-mediated auxin-responsive pathways, we further examined the co-expression relationships between the four candidate *QrIAA* genes and *QrARF* family members using the bulk RNA-seq dataset from adventitious root development stages (S10 and S11 Tables). Among the four candidate *QrIAA* genes, *QrIAA14-1* exhibited the highest weighted degree in the *QrIAA*-*QrARF* co-expression network (S4a Fig and S12 Table), indicating the strongest coordinated expression with *QrARF* members. Heatmap analysis further revealed the similar expression patterns between *QrIAA14-1* and seven *QrARF*s in R1, R2, R3, and R5 stages, including Qr02G001777 (*QrARF9*), Qr08G002297 *(QrARF18*), Qr11G000110 (*QrARF19-1*), Qr01G001280 (*QrARF10*), Qr02G000741 (*QrARF5*), Qr04G002344 (*QrARF8-2*), and Qr02G002584 (*QrARF8-1*) (S4b Fig and S11 Table). Notably, *QrARF8-1* displayed the prominent expression along the epidermis-to-root hair developmental trajectory (S4c Fig), suggesting it may represent a priority *QrARF* candidate within the QrIAA14-1-associated auxin regulatory module.

**Fig 6:**
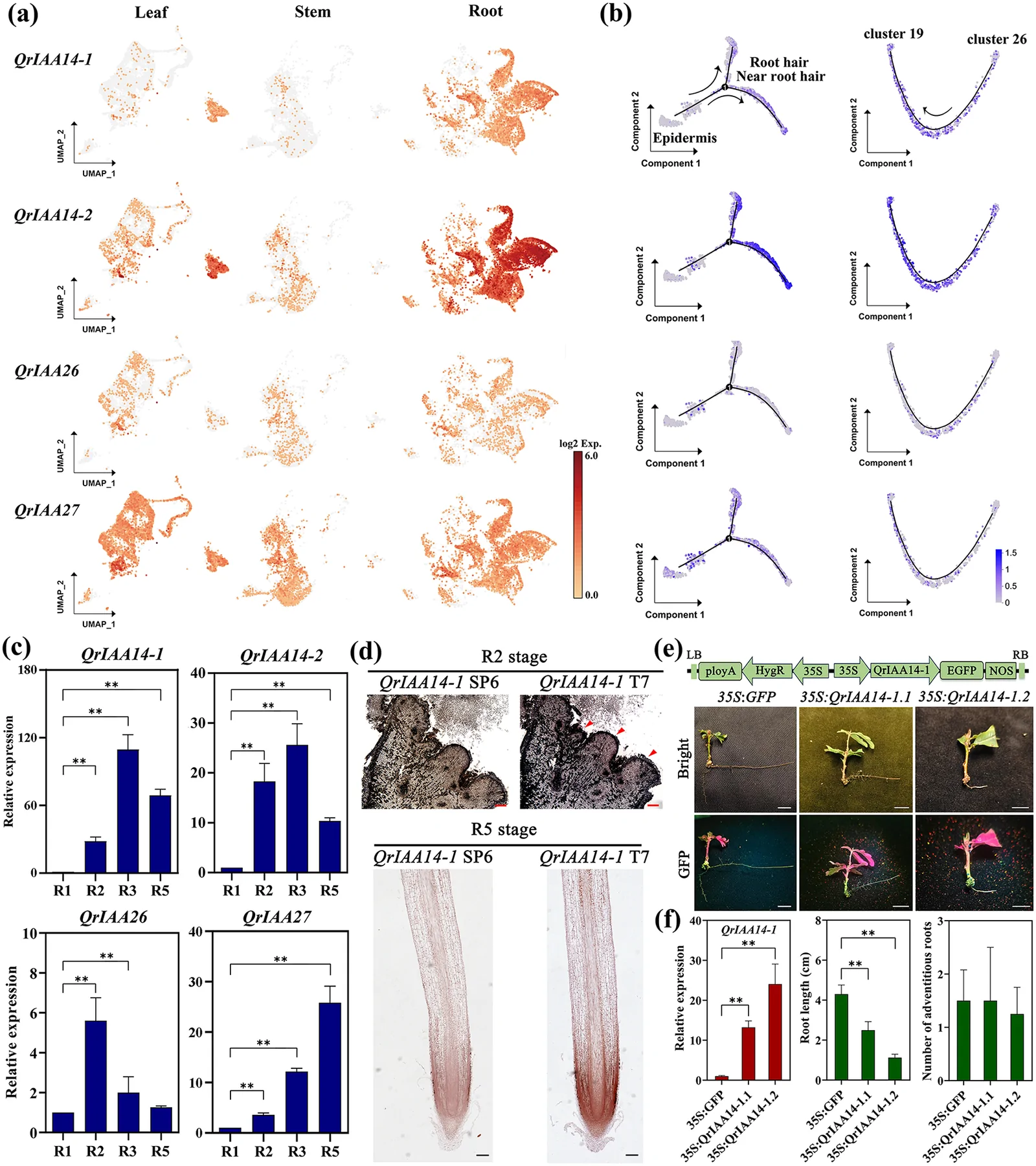
Identification and functional analysis of *QrIAA14-1* during adventitious root differentiation. a: UMAP visualization analysis of four candidate genes in three oak organs. b: Pseudo-time analysis of four candidate genes in the three cell types involved in root elongation growth. c: RT-qPCR analysis of four candidate genes during oak root development. d: Spatial expression patterns of *QrIAA14-1* revealed by RNA *in situ* hybridization in the callus tissue at R2 stages and adventitious roots at R5 stages. SP6 indicates the negative control using sense probes, whereas the T7-transcribed antisense probes were synthesized to detect *QrIAA14-1* transcripts, red arrows indicate the positive *QrIAA14-1* hybridization signals detected in the root primordia at the R2 stage, and *QrIAA14-1* hybridization signals were dominantly detected in the root cap region of the T7 antisense-probe samples at the R5 stage, whereas weaker staining was observed in other root regions as well as in the SP6 sense-probe control, the scale bar = 200 μm. e: Overexpression assays revealing the function of *QrIAA14-1* in oak root development, EGFP fluorescence was identified using the LUYOR-3415RG fluorescent protein lamp, scale bar = 1 cm. f: The relative expression of *QrIAA14-1*, adventitious root length and adventitious root numbers investigation in *35S:GFP* and transgenic lines. Error bars represent mean ± SD from four plantlets of each line, and two asterisks indicated very significant difference (*p* < 0.01) between the *35S:GFP* and *QrIAA14-1* transgenic lines.

To further prioritize the candidate AUX/IAA regulators governing root development in *Q. robur*, we investigated the expression patterns of the four *QrIAA* candidates at the single-cell level (Fig 6). The results showed that *QrIAA14-1* and *QrIAA14-2* were predominantly expressed in root cell clusters (Fig 6a), whereas *QrIAA26* and *QrIAA27* did not exhibit obvious differences among the clusters of root, stem, and leaf. Furthermore, within the epidermis-to-root hair developmental trajectory, *QrIAA14-1* and *QrIAA14-2* were preferentially enriched in root hair and near root hair cells (Figs 4i and 6b), with relatively lower abundance in the epidermal cell states, and both were significantly up-regulated during root cap development (Fig 6b). These findings indicated that *QrIAA14* may play important roles in root formation and differentiation in *Q. robur*.

In addition, RT-qPCR analysis showed that *QrIAA14-1*, *QrIAA14-2*, *QrIAA26*, and *QrIAA27* significantly upregulated during oak root development process (Fig 6c), which was consistent with RNA-seq findings (Fig 5d). Notably, the expression abundance of *QrIAA14-1* increased more than 100-fold at the R3 stage compared with the R1 stage, suggesting a prominent regulatory role in adventitious root development. Moreover, RNA *in situ* hybridization further revealed that *QrIAA14-1* was preferentially expressed in the root primordium-associated tissues at the R2 stage and remained highly abundant in the oak root cap region of R5 stage (Fig 6d), which was consistent with the scRNA-seq-based significant enrichment of *QrIAA14-1* in root cap-associated populations (Fig 4i). Furthermore, we generated *QrIAA14-1* overexpression lines, in which the expression levels of *QrIAA14-1* were approximately 20-fold higher than those in *35S:GFP* lines (Fig 6e and 6f). Notably, the *QrIAA14-1* overexpression lines exhibited about 64.46% decrease in adventitious root length at 30 days (*p* < 0.001, Student’s t-test), indicating that *QrIAA14-1* negatively regulates adventitious root elongation. Taken together, these evidences consistently highlighted *QrIAA14-1* as a transcriptional repressor negatively associated with adventitious root development in *Q. robur*.

## Discussion

Fagaceae is a globally significant plant family, whose members play essential roles in forest ecosystems and provide valuable sources for food and timber production [30]. Elucidating the differentiation trajectories of vegetative organs is not only fundamental to understand the regulatory networks governing plant development but also crucial for enhancing breeding efficiency in oak species. In recent years, extensive genomic and transcriptomic studies have linked the specific genes to ecological adaptation and physiological traits in oaks [35]. However, the molecular mechanisms by which distinct cell types are specified and coordinated during the development of vegetative organs remain largely unclear. The scRNA-seq has emerged as a powerful approach to resolve cellular heterogeneity and reconstruct developmental trajectories at high resolution [36], and it has been successfully applied to investigate organ development in a variety of herbaceous and woody plants. Nevertheless, most previous studies have focused on individual organs [21,27–29], which restricts the ability to compare organ-specific cell populations and identify divergent regulatory programs underling tissue-specific differentiation. To address these knowledge gaps, we constructed a comprehensive single-cell transcriptomic atlas encompassing leaf, stem, and root tissues of *Q. robur*. By profiling multiple vegetative organs within a unified analytical framework, this study provides an organ-resolved view of cell-type composition and differentiation trajectories, thereby providing a valuable resource and working framework for future mechanistic studies of vegetative organ development in oaks.

Furthermore, protoplast dissociation is a critical prerequisite for successful plant scRNA-seq profiling, yet this step remains particularly challenging in woody species because of secondary cell wall thickening, lignification, and tissue-level heterogeneity [16,36]. Previous studies have shown that optimized protoplast isolation strategies can resolve diverse cell populations in *Populus trichocarpa* [22,23,36], *Eucalyptus grandis* [24], *Taxus mairei* [37], and *Cunninghamia lanceolata* [38], supporting the feasibility of single-cell profiling for woody plant developmental researches [17]. Consistently, the successful dissociation of leaf, stem, and root tissues in the present study indicates that the optimized protoplast isolation strategy captured viable and transcriptionally informative cell populations in *Q. robur*. Nevertheless, mature lignified vascular cells and thick-walled fiber cells may be less efficiently recovered during enzymatic dissociation because of extensive secondary wall deposition and lignin accumulation, potentially leading to relatively lower representation in protoplast-derived scRNA-seq datasets [39]. In addition, protoplast isolation may perturb native transcriptional states by triggering wound-, osmotic stress-, and digestion-related responses, and these effects may difference among leaves, stems, and roots [16,40]**.** Therefore, scRNA-seq findings are more informative when evaluated together with complementary molecular, physiological, and functional evidence [41,42]. In this study, we integrated scRNA-seq with bulk RNA-seq, phytohormone profiling, RT-qPCR, RNA *in situ* hybridization, and transgenic validation, which collectively enhanced the reliability of our cell-type annotations and candidate gene identifications. Future studies could incorporate the cell-type-resolved spatial validations, loss-of-function analyses, and protein interaction assays, which will help further refine our understanding of the regulatory mechanisms underlying organ development in oaks.

The oak leaf-specific clusters resolved seven major cell types and delineated organ-specific transcriptional programs underlying epidermal and mesophyll lineages. Among them, the epidermal-guard cell represented a prominent differentiation trajectory, revealing a gradual and coordinated transcriptional transition from epidermal precursors to mature guard cells [43]. Notably, transcriptional clustering along the pseudotime axis uncovered four distinct gene modules with cell-type-specific activation patterns (Fig 2d). Genes within the guard cell-dominant module were significantly enriched in glycolysis, carbon fixation, and cutin and wax biosynthesis pathways (Fig 2e), consistent with the high energetic demand and structural specialization required for stomatal function [44]. Guard cells are specialized epidermal cells that flank the stomatal pore and dynamically respond to environmental and endogenous cues to regulate gas exchange and water balance [45,46]. Previous studies have demonstrated that *EPF* family are key regulators of stomatal development and density [47], and can participate in stress-responsive regulatory networks through interactions with transcription factors [48,49]. In addition, cyclic nucleotide-gated channels (*CNGC*s) function as central regulators of Ca² ⁺ signaling during stomatal movement [50]. Consistently, *EPF*- and *CNGC*-related genes were specifically enriched in oak guard cells, supporting their conserved roles in stomatal patterning and signal transduction while suggesting their involvement in guard cell maturation in this woody species [44]. Moreover, phytohormone signaling also appeared to contribute to epidermal lineage progression. Cytokinin signaling has been shown to modulate epidermal cell composition and promote stomatal development through the regulation of asymmetric cell division via SPEECHLESS [51–54]. In this study, three *AHP* genes, which encode primary phosphor-transfer proteins in cytokinin signaling, were enriched in both epidermal and guard cell clusters (S4 Table), indicating active cytokinin signal transduction during the transition from epidermal identity to guard cell fate. Collectively, these findings suggest that stomatal development in oak is governed by coordinated metabolic activation, conserved peptides, and Ca² ⁺ -mediated signaling pathways, and hormone-dependent regulatory programs, providing a refined view of epidermal lineage specification in a woody plant species.

As one of the most economically woody species, oak stems are highly valued for their superior mechanical strength and durability, making it essential to elucidate the genetic and developmental mechanisms governing secondary growth and wood formation. While stem cell differentiation and vascular patterning have been extensively characterized in herbaceous plants and crops [55,56], comparable analyses in woody perennials remain technically challenging due to highly secondary thickening and lignification, which severely limit efficient single-cell dissociation [22,36]. By applying an optimized protoplast strategy [36], we successfully resolved multiple stem-specific vascular populations, including cambium, xylem, xylem parenchyma cells, fiber cells, phloem, and sieve elements, thereby capturing the cellular complexity underlying secondary growth in oak. Additionally, pseudotime trajectory analysis reconstructed the differentiation branches of xylem, xylem parenchyma, and fiber cells. Genes enriched in the xylem cells were primarily associated with secondary metabolite biosynthesis pathways, consistent with the active lignification process that defines functional xylem differentiation [57]. Furthermore, the core genes in lignin biosynthesis pathways, including *PAL2*, *C4H*, and *CAD9*, exhibited fiber cell-specific upregulation, along with several peroxidases (*PER*s) required for oxidative lignin polymerization [58,59], these results suggested a tightly regulated transcriptional cascade driving extensive secondary wall thickening for mechanical strengthening in oak stems [60]. Meanwhile, the DEGs of xylem parenchyma cells were predominantly enriched in protein turnover and phytohormone signal transduction pathways, indicating a regulatory and homeostatic role within the vascular system. The identification of auxin- and cytokinin-responsive genes within this lineage implies that phytohormonal coordination may contribute not only to cambial activity but also to downstream vascular specialization [61]. Interestingly, consistent with previous studies [24,36], we detected enrichment of photosynthesis-related genes within some stem clusters, supporting the photosynthesis in chlorophyll-containing plastids of woody tissues [24]. This metabolic feature may provide supplementary carbon assimilation to sustain the high biosynthetic demand associated with secondary wall deposition [62]. Collectively, these findings indicate that vascular differentiation in oak stems involves lineage-specific metabolic activation, coordinated secondary wall biosynthesis, and hormone-associated regulatory networks. This integrated single-cell transcriptional framework refines our understanding of secondary growth in woody plants and highlights the cellular complexity underlying stem thickening and mechanical reinforcement in oaks.

Moreover, roots serve as the primary interface between plants and their environment [21], and their continuous growth is maintained by stem cell populations in the apical meristem that generate diverse cell types through coordinated differentiation programs [63]. Although root development has been extensively characterized in herbaceous species [21,28,63], knowledge of cell-type specification and transcriptional regulation remains limited in woody perennials [17,19]. In this study, we captured a well-defined continuum of cell types from meristematic to mature root tissues (Fig 4), revealing progressive transcriptional transitions in oak roots [19]. Recent studies have shown that root hairs are specialized epidermal cells that contribute to water and nutrient uptake as well as root-microbiome interactions [64,65]. Given the fundamental roles of root cap and root hair cells in morphogenesis and nutrient acquisition [66,67] and closely associated with root tip function and epidermal differentiation, therefore the pseudotime analysis focused on these lineages to resolve their differentiation dynamics. Notably, DEGs associated with auxin signaling were prominently enriched along the root developmental axis, highlighting hormone-mediated regulation as central driver of root cell fate specification. To integrate phytohormone profiles with transcriptional dynamics, we compared endogenous IAA accumulation with the expression patterns of AUX/IAA genes during adventitious root development. Interestingly, the endogenous auxin concentration reached its maximum at the R5 stage, while the *QrIAA14-1* was significantly enriched in root primordium callus tissue and root tips based on RNA *in situ* hybridization assays, and its expression abundance of *QrIAA14-1* was predominately upregulation during the oak root development process, especially adventitious root differentiation stage (R3 stage). Many previous studies have reported negative regulatory roles of *IAA4/11/12/19/28/14* genes in root development [6,7,9–11,68], although positive regulatory functions have also been described in *OsIAA13* and *PheIAA17* [12,14]. Consistent with these findings [7,11], we observed upregulation of *QrIAA* genes in root cap and root hair cell populations, and the overexpression of *QrIAA14-1* significantly inhibited the adventitious root elongation in oak. These findings suggested that the auxin levels and AUX/IAA-mediated repression may together reflect a feedback-associated regulatory response that modulates auxin signaling during adventitious root initiation and elongation [69], which may imply a potential molecular network contributing to the limited rooting capacity of *Q. robur* during tissue-culture propagation. Furthermore, numerous studies have demonstrated that the ARF-IAA module mediates auxin signaling [70,71], while AUX/IAA proteins function as transcriptional repressors that attenuate ARF activity [7,71]. In line with the previous studies, our co-expression analysis showed that *QrARF8-1* was prioritized as a potential *ARF* candidate within a putative QrIAA14-1-associated auxin regulatory module (S4 Fig). However, ARF proteins can be broadly classified into activator- and repressor-type members, and this functional distinction is largely associated with their phylogenetic grouping and the amino-acid composition of the middle region [72,73]. Previous studies shown that *ARF8-like* genes are positive regulators of early adventitious root formation and root developmental responses in *Arabidopsis* and *Populus* [74–76]. Consistently, *QrARF8-1* showed prominent expression along the epidermis-to-root-hair developmental trajectory in oak roots, suggesting it may be involved in auxin-associated root cell differentiation. Thus, future studies could employ the *QrIAA14-1* loss-of-function materials, ARF-AUX/IAA interaction assays, and auxin-responsive reporter analyses to clarify the underlying roles of *QrARF8-1*-*QrIAA14-1* module in responding to auxin signaling and regulating oak root development [7,63], which will provide a potential mechanistic insight into the hormone-centered regulatory framework of adventitious root regeneration in woody species [77].

## Conclusions

This study presents a comprehensive single-cell transcriptome atlas of major vegetative organs in *Q. robur*, which is the first systematic characterization of cell-type-specific gene expression in oak species. Thirty distinct cell clusters were identified across leaves, stems, and roots, enabling the dissection of organ-specific transcriptional programs and differentiation trajectories underlying guard cell differentiation, vascular formation, and root development. By integrating scRNA-seq with bulk RNA-seq, phytohormone investigation, RNA *in situ* hybridization, RT-qPCR, and transgenic assays, we identified auxin signaling as a crucial rooting regulator of oak root development. Notably, the overexpression of *QrIAA14-1* severely inhibited adventitious root elongation, providing a mechanistic insight into the developmental constraints associated with root development in oak species. Thus, our study not only provides a lot of cell-specific marker genes but also enriched our valuable resource for investigating the basic principles of vegetative development in oak species. Although several cell clusters remain unassigned, future integration with spatial transcriptomics is expected to further refine cell identity and spatial organization. Additionally, the functional dissection of these candidate key genes will enhance our understanding of cell fate determination and organ development at single-cell resolution in oaks.

## Materials and methods

### Oak materials and scRNA-seq library construction

*Quercus robur* seeds were collected from Linyuan Forest Farm, Xinshi District, Urumqi, Xinjiang, China (43.93°N, 87.46°E), and were used to establish aseptic *in vitro* cultures according to our previously published tissue-culture protocol [78]. Four-week-old tissue-cultured *Q. robur* plantlets (approximately 5–7 cm in height with 3–4 fully expanded leaves) were selected for scRNA-seq, which were maintained in a Conviron A1000 plant growth chamber (Conviron, Winnipeg, Canada) under a 16 h light (200 μmol·m^-^²·s^-^¹)/ 8 h darkness photoperiod, 24 ± 2 °C and 60% relative humidity. The leaves, stems and roots were excised and chopped into about 1 mm fragments, and protoplasts were isolated using a modified enzymatic digestion protocol [36]. Briefly, the three tissues were respectively incubated in digestion solution containing 1.5% (w/v) cellulase R10 (Yakult Pharmaceutical, Tokyo, Japan), 0.5% (w/v) macerozyme R10, 0.6 M mannitol, 20 mM KCl, 20 mM MES (pH 5.7), and 10 mM CaCl_2_ at 25 °C for 3–4 h with gentle shaking (60 rpm) [22]. The protoplast release was monitored under the microscope during enzymatic digestion. The digestion mixture was filtered through 40 μm cell strainers (Falcon, Corning, NY, USA) and washed twice with W5 solution (154 mM NaCl, 125 mM CaCl_2_, 5 mM KCl, 2 mM MES, pH 5.7). Cell viability was assessed by trypan blue exclusion (Solarbio, Beijing, China), and only samples with >85% viability was processed for library construction using 10x Genomics Single Cell workflow (Fig 1a). The final protoplast suspension was adjusted to an appropriate concentration for 10x loading (400–1200 protoplasts/μL). The cDNA libraries were estimated using an Agilent 2100 Bioanalyzer (Agilent Technologies, USA), and paired-end sequencing was performed by Majorbio Bio-Pharm Technology Co., Ltd. (Shanghai, China) on an Illumina NovaSeq 6000 platform (Illumina, USA) [23].

### scRNA-seq data pre-processing

The raw reads of scRNA-seq from roots, stems, and leaves were separately aligned to the *Q. robur* reference genome (Genome sequence archive number: CRA016241 and CRA016252) using the Cell Ranger pipeline (v2.0, 10x Genomics, USA) with default parameters, and gene-cell expression matrices were generated for each tissue and imported into Seurat (v4.0) for downstream analyses [79]. Genes expressed in fewer than three cells were removed. Cells expressing fewer than 200 or more than 9,000 genes were excluded, as were cells with fewer than 500 or more than 70,000 unique molecular identifiers (UMIs). Cells containing more than 5% mitochondrial transcripts were also filtered out. Potential doublets were identified and removed using Doublet Finder (v2.0.3) with a pN value of 0.25 [80]. The optimal pK was determined using the paramSweep_v3 function with the first 20 principal components, and the expected doublet rate was set to 7.5%, with the expected number of doublets adjusted to account for homotypic doublets. After filtering, the high-quality cells were counted from root, stem, and leaf tissues, respectively, and retained for further analyses. To enable integrated analysis across tissues, the three datasets were merged into a combined feature-barcode matrix using canonical correlation analysis (CCA) [81] and mutual nearest neighbors (MNN) [82] as implemented in Seurat (v3.1.0).

### Cell clustering and cell-type identification

Normalized gene expression data obtained using the LogNormalize function in Seurat (v4.0) were subjected to principal component analysis (PCA), and cell clusters were visualized using nonlinear dimensionality reduction [83]. Distinct cell populations were resolved in roots, stems, and leaves of *Q. robur* based on single-cell expression profiles. To achieve robust cell-type annotation, we integrated homologous gene information from *Arabidopsis thaliana* [63,84], soybean [85], wheat [86] and poplar [22,36]. We further referenced tissue-specific marker genes and their expression patterns from Plant Cell Marker (https://www.tobaccodb.org/pcmdb/homePage) and Plant scRNAdb (http://ibi.zju.edu.cn/plantscrnadb/) platforms to provided high-confidence annotation of cell identities across *Q. robur* organs, which were subsequently used for clustering and construction of the single-cell atlas (S4 Table). Additionally, all the marker genes used in this study were aligned against the *Populus trichocarpa* and *Arabidopsis thaliana* protein datasets using BLASTX to provide reference information for evaluating the marker gene functions (S4 Table).

### Pseudo-time trajectory analysis

Based on the annotated cell types, we further investigated the developmental dynamics of root, stem, and leaf cells at single-cell resolution using Monocle2 (v2.10.0) [63]. Raw data subsets from target clusters were extracted, and gene-wise expression dispersion was calculated across cells. Highly variable genes were then selected based on mean expression levels to infer developmental progression. Dimensionality reduction was carried out using the DDRTree algorithm [36], and cell trajectories were reconstructed with the order Cells function. Pseudo-time trajectories were visualized in Monocle2, and pseudo-time dependent genes were identified with BEAM for downstream visualization and interpretation [17].

### Differential expression and functional enrichment analysis of scRNA-seq dataset

Differentially expressed genes (DEGs) were identified for each target cell type using the Find Markers function implemented in Seurat (v4.0). Genes with an adjusted *P* value < 0.05 and |log_2_ fold change| > 0.25 were considered significantly differentially expressed [21]. Functional enrichment analysis of DEGs was performed based on the Kyoto Encyclopedia of Genes and Genomes (KEGG) database using the cluster Profiler R package (v4.0) [87] with a significant threshold as false discovery rate (FDR) < 0.05.

### Sequencing and analysis of bulk RNA-seq

Based on the morphological observation in this study, the adventitious root development of *Q. robur* plantlets can be divided into six stages: undifferentiated stage (R0), callus formation (R1), callus proliferation and root primordium initiation (R2), root differentiation and adventitious root tip appearance (R3), adventitious root elongation (R4), and adventitious root maturation (R5). The callus and/or adventitious root tissues from the basal rooting zone of plantlets at R1, R2, R3, and R5 stages were selected for bulk RNA-seq. For each stage, mixed tissues from five plantlets were collected with three biological replicates. Samples were immediately frozen in liquid nitrogen and stored at -80 °C until RNA extraction. Total RNA isolation, cDNA library construction and sequencing were performed by Novogene Bioinformatics Technology Co., Ltd. (Beijing, China) on the Illumina HiSeq 4000 platform as described in our previous studies [88].

Raw reads were processed to remove adapters and low-quality sequences (QPhred ≤ 20) to yield clean reads [89], which were aligned to the *Q. robur* reference genome. The gene expression levels were quantified with HTSeq and statistics as FPKM [90]. Fold change (FC) is the gene expression difference among the samples. Differentially expressed genes (DEGs) were identified using edgeR with thresholds of *p* < 0.05 and |log_2_FC| > 1 [91]. Kyoto Encyclopedia of Genes and Genomes (KEGG) enrichment analyses of DEGs were performed using KOBAS with significant enrichment defined as *p* < 0.05 [92]. Heatmaps of DEGs were visualized using TBtools software [93].

### Key genes identification related to root development

To identify key candidate genes, the DEGs in target enriched pathways were filtered with the criteria *p* < 0.05 and |log_2_FC| > 1. The expression data of these candidate genes were extracted to calculate Pearson correlation coefficients. Genes with correlation > 0.9 were retained to construct co-expression networks, which were visualized in Cytoscape [94]. To further explore the regulatory roles of key candidate DEGs identified from the co-expression network, their expression dynamics were mapped onto the scRNA-seq dataset of leaves, stems, and roots in *Q. robur* to examine the expression patterns across three organs. Meanwhile, the heatmaps were generated to visualize the expression of key candidate genes in all cell clusters.

For the ARF-AUX/IAA co-expression network, *QrARF* family members were identified using iTAK_v1.6a software (https://github.com/kentnf/iTAK/releases) [95]. The expression profiles of *QrARF* family members and selected *QrIAA* genes during oak root development were extracted from the bulk RNA-seq dataset and transformed as log_2_(FPKM+1). Pairwise Pearson correlation coefficients were calculated between *QrARF* and *QrIAA* genes. The weighted degree of each node was calculated as the sum of the absolute Pearson correlation coefficients of all retained edges connected to that node. Gene pairs with correlation > 0.6 were retained to construct co-expression networks, which were visualized in Cytoscape [94].

### Quantitative real-time PCR analysis

To validate the underlying roles of the candidate DEGs during adventitious root formation, the callus and/or adventitious root samples from the basal rooting zone of plantlets at R1, R2, R3, and R5 stages were analyzed by real-time quantitative PCR (RT-qPCR). Total RNA was extracted and reverse-transcribed into cDNA using the FastQuant RT Kit (Tiangen, Beijing, China), and the RT-qPCR was performed on a Bio-Rad CFX96 instrument (Bio-Rad, California, USA) [94]**.** Three biological replicates were included for each stage, and specific primers were designed using Primer Premier 5 (S13 Table). *QrFHY3* was used as an internal reference gene [96], and relative expression levels were calculated using the 2^-ΔΔCt^ method with the R1 stage as the control [89].

### RNA *in situ* hybridization

The R2 and R5 stages of root development were isolated to conduct *in situ* hybridization following the previous study [97]. Briefly, samples were fixed, dehydrated through an ethanol series, lightly stained with 0.1% eosin in 100% ethanol to visualize cell walls and cellular structures during tissue embedding and sectioning. The samples were then embedded in paraffin and sectioned for RNA *in situ* hybridization. Digoxigenin-labeled *QrIAA14-1* antisense probes were synthesized using T7 RNA polymerase with the DIG RNA Labeling Kit (SP6/T7; Sigma-Aldrich) according to the manufacturer’s instructions, whereas SP6-transcribed sense probes were used as negative controls. Primer sequences were listed in S13 Table. Hybridization signals were detected using a DIG Nucleic Acid Detection Kit (Roche, Germany) with an anti-digoxigenin alkaline phosphatase system and visualized with 20 μL NBT/BCIP per 1 mL TN buffer [21].

### Phytohormone quantification

The callus and/or adventitious root tissues were collected from the basal rooting zone of plantlets at the R1, R2, R3, and R5 stages were employed to quantify eight endogenous phytohormones using ultra-high-performance liquid chromatography-tandem mass spectrometry (HPLC-MS/MS) platform [98], containing indole-3-acetic acid (IAA), trans-zeatin (tZ), trans-zeatin riboside (tZR), salicylic acid (SA), jasmonic acid (JA), gibberellic acid 4 (GA_4_), abscisic acid (ABA), and 1-aminocyclopropane-1-carboxylic acid (ACC, the ethylene precursor). Approximately 100 mg of fresh sample was collected from four developmental stages with three biological replicates.

Phytohormones were extracted and purified using a modified method described by Xin et al. [98]. Briefly, frozen fresh samples were ground into a fine powder in liquid nitrogen and extracted with 1 mL of 90% methanol containing isotope-labeled internal standards, including D5-IAA, D6-ABA, D2-GA4, D5-JA, D5-tZ, D5-tZR, D4-SA, and D4-ACC, each at a final concentration of 2 ng/mL. The samples were extracted overnight at 4°C and then centrifuged at 4°C and 10,000 rpm for 15 min. The supernatant was collected and purified using mixed-mode solid-phase extraction according to the physicochemical properties of the target phytohormones. After purification, the eluted fractions were dried under a nitrogen stream and redissolved in a mobile-phase-compatible solvent before UPLC-MS/MS analysis.

Each sample was analyzed using a Xevo TQ-XS system equipped with an electrospray ionization source (Waters, USA). Chromatographic separation was performed on an ACQUITY UPLC HSS T3 column (2.1 × 100 mm, 1.8 μm; Waters, USA) maintained at 40°C. The mobile phase consisted of solvent A (0.1% formic acid in water) and solvent B (methanol), both formic acid and methanol were purchased from Sigma-Aldrich (St. Louis, MO, USA), and the flow rate was set at 0.3 mL min^−1^. The gradient elution program was as follows: 0–2 min, 2% B; 2–10 min, to 80% B; 10–12 min, 80% B; 12–13 min, to 2% B; 13–15 min, 2% B. The autosampler temperature was maintained at 4°C, and the injection volume was 10 μL. Detection was performed in multiple reaction monitoring (MRM) mode, and data analysis was performed using the spectrometer software (Masslynx v.4.2). One-way analysis of variance (ANOVA) followed by least significant difference (LSD) tests (*P* < 0.05) was applied to evaluate differences among the samples, and all figures were generated using GraphPad Prism 8 [99].

### Transgenic plant validation in oak seedlings

The coding sequence of *QrIAA14-1* (Qr06G000865) was amplified and cloned into the plant expression vector pCAMBIAsuper1300-EGFP. The positive construct was introduced into *Agrobacterium tumefaciens* strain EHA105 (Weidi Biotechnology, Shanghai, China) for plant transformation. Based on our previous oak tissue culture system [73], we further developed an *agrobacterium*-mediated transformation methods to generate *Q. robur* transgenic plantlets. The stem segments with axillary buds (1.5-2 cm) were used as explants, infected with *A. tumefaciens* EHA105 harboring pCAMBIAsuper1300-QrIAA14-1-EGFP at OD600 = 0.6 for 15 min, co-cultivated in dark for 3 days, then transferred to selection medium containing 25 mg/L hygromycin (Sangon Biotech, Shanghai, China) for 4 weeks to obtain resistant plantlets. Putative transgenic lines were screened based on hygromycin resistance and EGFP fluorescence using a portable fluorescent protein excitation lamp equipped with a GFP observation filter (3415RG; LUYOR, Joliet, IL, USA) [100,101], the wild-type plants transformed with the empty vector were used as negative controls. Two independent transgenic lines were investigated to explore the gene function, and four plantlets of each line were analyzed for phenotypic evaluation. The overexpression lines of *QrIAA14-1* were further confirmed by RT-qPCR assays. Root system architecture was documented by digital imaging, and adventitious root length was measured using ImageJ software (v.1.8.0).

## Supporting information

S1 Fig Cluster analysis of the single-cell dataset. (a) Correlation Heatmap of different cell clusters. The color represents the level of correlation between two clusters, with red indicating positive correlation and blue indicating negative correlation. (b) Statistical proportion of the clusters in three organs. The y-axis represents the proportion of cells in roots, stems, and leaves.
(DOCX)

S2 Fig The key DEGs identification from the pseudo-time analysis in oak stem. (DOCX)

S3 Fig The key transcriptional regulator identification of the two comparisons. The figure showed the TOP30 DEGs in each comparison, the bubble size represents the log2(fold change), and the red font highlights the *IAA* genes in TOP15 transcriptional regulator in each comparison.
(DOCX)

S4 Fig Integrated bulk RNA-seq and single-cell trajectory analyses identify QrIAA14-1-associated ARF candidates during oak root development. (a) Co-expression network of the four candidate *QrIAA* genes and *QrARF* family members based on bulk RNA-seq data from root developmental stages. Node and label sizes represent the weighted degree of each gene in the network, the edge thickness indicates the strength of Pearson correlation-based co-expression relationships. (b) Heatmap showing the scaled expression profiles of four *QrIAA* genes and *QrARF* family members in R1, R2, R3 and R5 stages, revealing distinct stage-dependent expression dynamics. (c) Single-cell trajectory plots showing the expression patterns of the representative *ARF* genes associated with *QrIAA14-1* in the epidermis-to-root hair developmental trajectory.
(DOCX)

S1 Table Quality control of single-cell dataset. (XLSX)

S2 Table The Statistical analysis of cell quantity in each cluster. (XLSX)

S3 Table The cluster identification based on the cell number in each organs. (XLSX)

S4 Table The list of marker genes used in this study. (XLSX)

S5 Table The key DEGs enrichement from pseudo-time analysis of guard cell and epidermis in oak leaves. (XLSX)

S6 Table The key DEGs enrichement from pseudo-time analysis of xylem related cells in oak stem. (XLSX)

S7 Table Quality control of RNA-seq dataset for root development process. (XLSX)

S8 Table Alignment of clean reads to the reference genome. (XLSX)

S9 Table DEGs significantly enriched in phytohormone signal transduction pathways based on RNA-seq data. (XLSX)

S10 Table The expression abundance of four candidate *QrIAA* genes and *QrARF* family members from bulk RNA-seq datasets. (XLSX)

S11 Table The network profile between four candidate *QrIAA* genes and *QrARF* family members base on bulk RNA-seq datasets. (XLSX)

S12 Table The node parameters of the network profile between four candidate *QrIAA* genes and *QrARF* family members. (XLSX)

S13 Table Primers for RT-qPCR and RNA *in situ* hybridization of key genes selected in this study. (XLSX)
