# Supplementary material for: Multi-organ single-cell transcriptomic atlas identifies QrIAA14 as a candidate negative regulator of adventitious root development in Quercus robur
Source: PLoS Genet. 2026 Sep 1;22(9):e1012300. doi: 10.1371/journal.pgen.1012300 (PMC13552951; doi:10.1371/journal.pgen.1012300)
Supplement: Supplementary file 1 [file pgen.1012300.s001.docx]

***Supplementary Figures***

**S1 Fig. Cluster analysis of the single-cell dataset.** (a) Correlation Heatmap of different cell clusters. The color represents the level of correlation between two clusters, with red indicating positive correlation and blue indicating negative correlation. (b) Statistical proportion of the clusters in three organs. The y-axis represents the proportion of cells in roots, stems, and leaves.
