# Supplementary material for: Multi-organ single-cell transcriptomic atlas identifies QrIAA14 as a candidate negative regulator of adventitious root development in Quercus robur
Source: PLoS Genet. 2026 Sep 1;22(9):e1012300. doi: 10.1371/journal.pgen.1012300 (PMC13552951; doi:10.1371/journal.pgen.1012300)
Supplement: Supplementary file 3 [file pgen.1012300.s003.docx]

***Supplementary Figures***

**S3 Fig. The key** **transcriptional regulators identification of the two comparisons.** The figure showed the TOP30 DEGs in each comparison, the bubble size represents the log2(fold change), and the red font highlights the IAA genes in TOP15 transcriptional regulator in each comparison.
