# Supplementary material for: Multi-organ single-cell transcriptomic atlas identifies QrIAA14 as a candidate negative regulator of adventitious root development in Quercus robur
Source: PLoS Genet. 2026 Sep 1;22(9):e1012300. doi: 10.1371/journal.pgen.1012300 (PMC13552951; doi:10.1371/journal.pgen.1012300)
Supplement: Supplementary file 4 [file pgen.1012300.s004.docx]

***Supplementary Figures***

**S4 Fig. Integrated bulk RNA-seq and single-cell trajectory analyses identify QrIAA14-1-associated ARF candidates during oak root development.** (a) Co-expression network of the four candidate *QrIAA* genes and *QrARF* family members based on bulk RNA-seq data from root developmental stages. Node and label sizes represent the weighted degree of each gene in the network, the edge thickness indicates the strength of Pearson correlation-based co-expression relationships. (b) Heatmap showing the scaled expression profiles of four *QrIAA* genes and *QrARF* family members in R1, R2, R3 and R5 stages, revealing distinct stage-dependent expression dynamics. (c) Single-cell trajectory plots showing the expression patterns of the representative ARF genes associated with *QrIAA14-1* in the epidermis-to-root hair developmental trajectory.
