# Supplementary material for: Multi-organ single-cell transcriptomic atlas identifies QrIAA14 as a candidate negative regulator of adventitious root development in Quercus robur
Source: PLoS Genet. 2026 Sep 1;22(9):e1012300. doi: 10.1371/journal.pgen.1012300 (PMC13552951; doi:10.1371/journal.pgen.1012300)
Supplement: Supplementary file 6 [file pgen.1012300.s020.docx]

***Point-by-point response***

**Reviewers' comments:**

**Reviewer #1:**

They conducted single-cell RNA-seq in the leaves, stems, and roots of *Quercus robur* to identify the differences among the three organs. In addition, they conducted bulk RNA-seq at different stages of adventitious root regeneration, from callus on the stem, to explore the suppressive factors of root development in *Q. robur*. They also conducted phytohormone analysis, *in situ* hybridization, and morphological analysis using an overexpression line to confirm their sequence data. The extensive experiments provide a valuable dataset and suggest that *QrIAA14-1* repressed adventitious root initiation and development in *Q. robur*. While the study provides a wide dataset on woody plants, especially oak, there are several areas where the material and methods section, data presentation, and the strength of the conclusions need clarification and improvement.

**Major points:**

1. The use of the word "critical" in the title is too strong to describe *QrIAA14-1* as a repressor of adventitious root formation. The authors described that *QrIAA14-1* suppresses root initiation and development in the manuscript, but roots are still induced in the *QrIAA14-1* overexpression line. This data suggests that *QrIAA14-1* may play a partial role in suppressing root development rather than strictly repressing root initiation. I recommend changing the title and some parts of the manuscript. It would also be helpful to show a time-course analysis of adventitious root numbers and lengths in the wild-type and *QrIAA14-1* overexpression lines, or to provide data on the number of adventitious roots in a *Qriaa14-1* loss-of-function mutant, which would support the conclusion.

**Response**: Thank you for this important and constructive suggestion. We agree that the term “critical” may overstate the role of *QrIAA14-1*, especially because adventitious roots were still induced in the *QrIAA14-1* overexpression lines. Therefore, we have revised the title and the relevant descriptions in the Abstract, Results, Discussion, and Conclusion to accurately describe the role of *QrIAA14-1* in adventitious root development.

To further clarify the phenotype of *QrIAA14-1* overexpression lines, we added an additional independent overexpression line and performed quantitative analyses of adventitious root length and adventitious root number in the revised manuscript (**Fig. 6e-f**). The results showed that *QrIAA14-1* overexpression significantly reduced adventitious root length, whereas the number of adventitious roots was not significantly different from that of the control. These additional data support a more precise conclusion that *QrIAA14-1* mainly affects adventitious root development and elongation, rather than strictly preventing adventitious root initiation. The corresponding Results section and figure legend have been provided in **Lines 349-355** and **Lines 820-824** of the revised manuscript.

2. In line 246, the authors described that primary root tips appeared in R3 stage. However, it is difficult to recognize the roots or callus in R3 stage from the current images (Fig 5a). Please provide higher-resolution images or a magnified image for R3 stage in Fig. 5a.

**Response**: Thank you for this helpful suggestion. We have added a magnified view of the basal region at the R3 stage to better display the root differentiation process (**Fig. 5a**). The newly emerged adventitious root tips are now indicated with red arrows to facilitate visual recognition. We have also revised the corresponding description in the Results section to use “adventitious root tips” instead of “primary root tips” for more accurate terminology. The details are provided in **Line 255-256** of the revised manuscript.

3. The Materials and Methods section lacks sufficient explanation; please clarify the methods to ensure reproducibility.

- About materials: Please specify the source or origin of the Q. robur plants. For the "4-week-old" plants (line 458), clarify whether this is from seed germination or shoot meristem culture. Additionally, describe the growth conditions (soil or culture media, type of growth chamber).
- Please provide the source of the reference genome (line 473).
- Please describe the methods of RNA isolation and cDNA reaction in RT-qPCR (line 544).
- Please describe the methods of phytohormone analysis: extraction and purification protocols, and specify the company of the instrument. Importantly, clarify which exact plant parts (whole plants, specific tissues, callus, or root parts) were collected for the 100 mg samples.

**Response**: Thank you for this helpful suggestion. We have revised the Materials and Methods section to provide additional details.

- We have added information on the source and culture conditions of the *Q. robur* materials. The seeds were collected from Linyuan Forest Farm, Xinshi District, Urumqi, Xinjiang, China, and were used to establish tissue-cultured plantlets according to our previously published protocol. The tissue-cultured plantlets maintained in a Conviron A1000 plant growth chamber (Conviron, Winnipeg, Canada) under a 16 h light (200 μmol·m-²·s-¹)/8 h darkness photoperiod, 24±2°C and 60% relative humidity. The details are provided in **Lines 508-513** of the revised manuscript.
- The source of reference genome had been added with the Genome sequence archive number: CRA016241 and CRA016252 in the resubmission. The details are provided in **Line 530-532** of the revised manuscript.
- We have added the methods of RNA isolation and cDNA synthesis used in RT-qPCR analysis. The details are provided in **Lines 617-619** of the revised manuscript.
- We have rewritten the phytohormone quantification method to include additional details on sample collection, extraction, and purification procedures, UPLC–MS/MS instrumentation, chromatographic conditions, and data processing. We have also clarified that callus and/or adventitious root tissues were collected from the basal rooting zone of plantlets at the R1, R2, R3, and R5 stages for phytohormone analysis. The details are provided in **Lines 632-650** of the revised manuscript.

4. The developmental states of the plants used for each stage appear inconsistent in Fig. 5a. For instance, R0 plants lack internodes, whereas others have multiple internodes. Leaves are cut in half in R0–R3, but R4 and R5 differ. The R2 callus appears larger than that in R3, and leaf numbers vary. If whole plants were sampled for bulk RNA-seq, RT-qPCR, and phytohormone analyses, these morphological inconsistencies would affect the results. Please clarify which tissues or whole plants were sampled and describe how these sample variations were controlled.

**Response:** Thank you for your constructive comments. We would like to clarify that Fig.5a was intended to show representative morphological features of adventitious root development, especially in the basal rooting region, rather than to indicate that whole plantlets were sampled for downstream analyses. For bulk RNA-seq, RT-qPCR, and phytohormone quantification, whole plantlets were not collected. Instead, callus and/or adventitious root tissues were specifically sampled from the basal rooting zone of plantlets at the R1, R2, R3, and R5 stages. Each biological replicate consisted of pooled basal rooting tissues from five plantlets at the same developmental stage, which helped reduce variation among individual plantlets. The developmental stage was defined primarily according to the status of callus formation, root primordium initiation, adventitious root tip appearance, adventitious root elongation, and adventitious root maturation in the basal region.

We also clarified that the upper leaves were partially trimmed during early rooting culture because the original leaves were relatively large, whereas the leaves observed at later stages were newly developed during the rooting process (we have cited our previous study to clarify the detailed information, Wang, et al., Life). Although aboveground tissues may influence the physiological status of plantlets, they were not included in the sampled materials for transcriptomic or phytohormone analyses. Therefore, the observed variation in leaf size, leaf number, or internode development in the images did not directly affect the composition of the sampled tissues used for molecular analyses of basal adventitious root development. We have clarified the sampling strategy and tissue types in the revised Materials and Methods section and Fig.5a legend. The details are provided in **Lines 579-581, Lines 615-617, Lines 633-635**, **Lines 795-799** of the revised manuscript.

5. Interpreting the results of *in situ* hybridization (Fig. 6d) was difficult.

- The labels "mock" and "*QrIAA14-1*" should be corrected to standard terminology, such as "sense" and "anti-sense" probes.
- The signals in both the R2 and R5 stages are difficult to distinguish. In addition, please explain what the red arrows indicate.
- Please provide a diagram or describe the exact sectioning plane or position used for the R2 stage, as it is currently difficult to identify the tissue part.

**Response**: Thank you for this helpful suggestion. We have revised the Materials and Methods section and Fig. 6d to provide additional details and improve the interpretation of the RNA in situ hybridization results.

- We have revised the labels and legend of **Fig.6d** to clarify the information of sense and anti-sense probes. Specifically, the SP6-transcribed sense probe was used as the negative control, whereas the T7-transcribed antisense probe was used to detect QrIAA14-1 transcripts. This information has also been clarified in the Materials and Methods section. The details are provided in **Lines 626-630** and **Lines 815-819** of the revised manuscript.
- Compared with the SP6 sense-probe control, positive QrIAA14-1 hybridization signals were detected in the T7 antisense-probe samples, particularly in the root primordia at the R2 stage and in the root tip region at the R5 stage. The red arrows indicate obvious positive *QrIAA14-1* hybridization signals detected in the root primordia at the R2 stage. We have revised the Results section and the legend of Fig.6d to clarify the information.
- We have also clarified the sample information of R2 and R5 stages in the legend of Fig.6d. RNA *in situ* hybridization was conducted using the callus tissue from basal rooting zone of R2 stages and adventitious roots at R5 stages. The details are provided in **Lines 815-819** of the revised manuscript.

6. In Fig 6d, the differences between the *p35S:GFP* and *p35S: QrIAA14-1:GFP* signals are not visually recognizable. The high background signal and prominent artifacts complicate interpretation, and there are concerns about the possibility of overexposure. Furthermore, the imaging conditions (e.g., microscope and exposure time) are not provided in the Materials and Methods. Please clarify the exact purpose of this image comparison in the text and provide cleaner images with appropriately optimized exposure settings. Additionally, please include imaging parameters in the Methods section.

**Response:** Thank you for this helpful comment. Because the transgenic oak plantlets were relatively large, we used a portable fluorescent protein excitation lamp for rapid screening of EGFP-positive plantlets. In this study, the putative transgenic lines were first screened based on hygromycin resistance and then examined for EGFP fluorescence using a portable fluorescent protein excitation lamp. The portable fluorescent excitation lamp has been used in recent plant studies for rapid screening and visualization of fluorescent marker-positive tissues (He *et al.*, Plant Physiol. 2024; Zhang *et al.*, Nat. Commun., 2025; Bai *et al.*, Nat. Commun., 2026). Because this device was used as a qualitative screening tool rather than a fluorescence microscope, the microscope exposure time was not applicable. Thus, the overexpression of *QrIAA14-1* was further confirmed by RT-qPCR, and the functional conclusions were based on *QrIAA14-1* transcript abundance and adventitious root phenotypic measurements. We have revised the Materials and Methods section and the legend of Fig.6e in the revised manuscript. The details are provided in **Lines 661-664** and **Lines 820-821** of the revised manuscript.

7. Lines 230-231 mentioned that conducting KEGG enrichment analysis focuses on the root cap and near root hair cells. Since the study focuses on adventitious root initiation and development, focusing on the root meristem would seem more appropriate. Please provide a scientific rationale for choosing the root cap and root hair cells over the meristematic zone.

**Response:** Thank you for this insightful comment. We agree that the root meristem is central to adventitious root initiation and continuous root growth. In this study, the KEGG enrichment analysis was performed on root cap and epidermal-lineage populations, including near-root-hair and root hair cells, because these populations were clearly annotated in our dataset and are closely associated with root tip function and epidermal differentiation. Recent studies have shown that root hairs are specialized epidermal cells that contribute to water and nutrient uptake as well as root-microbiome interactions. Therefore, root cap and root hair-related epidermal-lineage populations provide informative cellular contexts for investigating hormone-associated regulatory programs during oak root development. We have revised the Results and Discussion sections to clarify why these cell populations were selected for KEGG enrichment analysis. The details are provided in **Lines 237-239** and **Lines 459-463** of the revised manuscript.

8. There are inconsistencies in the numerical data between the main text and the supplementary tables (e.g., line 115 mentions 6,892 cells, but the S2 table mentions 5,943 cells. Line 251: 44.17% as well). Please carefully review and correct all numerical data for consistency.

**Response:** Thank you for pointing out these inconsistencies. We sincerely apologize for the errors in the numerical descriptions in the original manuscript, these data have been corrected. We have carefully checked all numerical data in the main text, figures, and supplementary tables to ensure consistency throughout the revised manuscript. The details are provided in **Line 117** of the revised manuscript.

9. The statement “The leaf-derived cells were predominantly distributed in 17 clusters (Fig. 1c), with cluster 2, cluster 25, and cluster 27 consisting of more than 98%.” (lines 121–122) is unclear. Please clarify the denominator for this 98% and how this number was calculated.

**Response:** Thank you for this helpful comment. The “more than 98%” value refers to the fraction of leaf-derived cells within each indicated cluster, with the total number of cells in the corresponding cluster used as the denominator. Specifically, leaf-derived cells accounted for 98.11% of cells in cluster 2 (2,653/2,704), 100% in cluster 25 (123/123), and 99.04% in cluster 27 (103/104), as showed in **Table S2** and **Figure S1**. We have revised the corresponding sentences to clarify these values and the cellular composition of each cluster. The details are provided in **Lines 122-125, Table S2** and **Figure S1** of the revised manuscript.

**Minor points**

1. Please describe the company names for all key chemicals, kits, and equipment mentioned in the Methods.
2. Provide the full name of a gene only at its first mention in the text (e.g., lines 55-56, lines 529-530). Thereafter, consistently use the abbreviation.
3. Both "plant hormones" and "phytohormones" are used in the manuscript. Please choose one term and use it consistently throughout.
4. There are extra closing parenthese and a period ")." at line 121.
5. Please arrange the cluster numbers for each organ in Table S3 in numerical order to improve readability.

**Response to Minor points:** Thank you for these careful suggestions. We have carefully checked the manuscript and revised the corresponding issues as follows.

- We have added company names for all key chemicals, kits, and equipment mentioned in the Materials and Methods section.
- We have checked the full names and abbreviations of genes throughout the manuscript, and the full names are now provided at their first mention.
- We have checked these words throughout the manuscript to replace plant hormones using phytohormones.
- We have corrected the extra closing parenthesis and period in **Line 123**.
- We have re-arranged the cluster numbers for each organ in **Table S3** in numerical order to improve readability.

**Reviewer #2:**

This manuscript presents a comprehensive and high-quality single-cell transcriptomic landscape of Quercus robur. The authors have addressed the technical challenges associated with protoplast isolation in woody species and integrated scRNA-seq, bulk RNA-seq, and phytohormone profiling to identify QrIAA14-1 as a potential negative regulator of adventitious rooting. The study is timely, the methodology is sound, and the discovery of the IAA14-mediated inhibitory mechanism provides valuable insights into the vegetative propagation of forest trees. However, there still are some comments for the authors to revise the manuscript for publication.

**Major Points for the Authors:**

1. The authors rely heavily on Arabidopsis orthologs for cell-type annotation. I suggest providing more evidence or discussion to support whether these markers are consistently expressed in the corresponding tissues of Quercus. The authors may also consider to compare with available single-cell datasets from woody plants, such as Populus, to strengthen the reliability of cell-type annotation.

**Response:** Thank you for this constructive suggestion. In this study, the cell-type annotation was supported by integrated homologous gene information from multiple plant species, including Arabidopsis thaliana, soybean, wheat, and the woody model species Populus trichocarpa. We also referenced tissue-specific marker genes and their expression patterns from Plant Cell Marker and Plant scRNAdb. In the revised manuscript, all marker genes used in this study were aligned against the *Populus trichocarpa* and *Arabidopsis thaliana* protein datasets using BLASTX, and the corresponding homologous gene IDs and E-values were provided in **Table S4**. The details are provided in **Lines 555-557** of the revised manuscript.

2. Although overexpression experiments have clearly demonstrated that QrIAA14-1 inhibits root elongation in oak, AUX/IAA proteins generally function by interacting with ARF transcription factors to repress auxin-responsive transcriptional activation. To determine whether a similar pathway may carry out in Q. robur, the authors should perform a co-expression analysis using the scRNA-seq or bulk RNA-seq datasets. Specifically, the authors are encouraged to identify which ARF members show coordinated expression patterns with QrIAA14-1, thereby highlighting the potential regulatory module involved in oak root development.

**Response:** Thank you for this constructive suggestion. We agree that AUX/IAA proteins generally modulate auxin-responsive transcription through associations with ARF transcription factors, and that identifying potential ARF candidates would strengthen the interpretation of the QrIAA14-1-associated auxin regulatory pathway.

To address this comment, we performed an additional co-expression analysis between the four candidate *QrIAA* genes and QrARF family members using the bulk RNA-seq dataset from root developmental stages, which identified several QrIAA14-1-associated QrARF candidates, including *QrARF9*, *QrARF18*, *QrARF19-1*, *QrARF10*, *QrARF5*, *QrARF8-2*, and *QrARF8-1*. Among these ARF candidates, *QrARF8-1* showed prominent expression along the epidermis-to-root hair developmental trajectory in the single-cell dataset, suggesting that it may represent a priority ARF candidate within the putative QrIAA14-1-associated auxin regulatory module.

These analyses have been added as a **new supplementary figure, Fig. S4**. The corresponding datasets have also been provided in **new Tables S10-12**, including the gene expression profile, co-expression network dataset, and weighted degree values for each gene. The Materials and Methods, Results and Discussion sections have been revised in the **Lines 319-330, Lines 480-483,** and **Lines 605-612** of the revised manuscript.

3. In Figure 5, the correlation between endogenous IAA content and QrIAA14-1 expression is particularly interesting. The authors should further discuss the potential feedback loop mechanism to explain how the oak tissues balance elevated auxin levels with expression of the AUX/IAA repressor during root initiation and differentiation.

**Response:** Thank you for this helpful suggestion. We agree that the temporal relationship between endogenous auxin accumulation and *QrIAA14-1* expression is important for understanding the potential role of AUX/IAA-mediated repression during adventitious root development.

We have rewritten the relevant section of Discussion to address this point. Given the repressive role of AUX/IAA proteins in auxin signaling, we further discuss that this pattern may reflect a feedback-associated regulatory response during the transition from root primordium initiation to root elongation. We also added a future perspective that auxin-responsive reporters, *ARF* target-gene analyses, and auxin sensitivity assays could further clarify the potential mechanism by which *QrIAA14-1* responds to auxin and regulates oak adventitious root development. The details are provided in **Lines 465-487** of the revised manuscript.

4. In Discussion section, the author should further illustrate the key role of IAA14-1 in auxin signaling pathways, particularly the potential regulatory mechanisms underlying the poor rooting ability in Q. robur.

**Response:** Thank you for this helpful suggestion. We have revised the Discussion to further illustrate the role of *QrIAA14-1* within the auxin signaling pathway. Specifically, we discussed the ARF-AUX/IAA regulatory framework and described *QrIAA14-1* as a candidate AUX/IAA repressor associated with adventitious root development. We further connected endogenous auxin accumulation, *QrIAA14-1* induction, RNA *in situ* hybridization signals, overexpression phenotypes, and the newly added QrIAA-QrARF co-expression analysis to propose that QrIAA14-1-associated AUX/IAA repression may represent one potential molecular component contributing to the limited rooting capacity of *Q. robur* during tissue-culture propagation. The details are provided in **Lines 453-487** of the revised manuscript.

5. Please provide more details on the optimized protoplast strategy mentioned in line 380. For woody plants, the enzymatic digestion time and vacuum infiltration steps are critical for reproducibility.

**Response:** Thank you for this helpful suggestion. We have added more details on the optimized protoplast isolation strategy in the Materials and Methods section, including enzyme composition, digestion time, filtration, washing, and cell viability assessment.

In addition, we have revised the Discussion to provide more description of key technical considerations associated with protoplast dissociation in woody species, including digestion efficiency, lignified cell recovery, cell-type representation, and potential protoplasting-induced transcriptional perturbations. The details are provided in the revised Materials and Methods and Discussion section, **Lines 516-524** and **Lines 376-395**, respectively.

**Minor Points:**

1. Please check the manuscript for consistent gene nomenclature, including the use of italics for gene names and non-italics for proteins.

2. Please correct minor typographical errors throughout the manuscript. For example, in Line 469, “with he expected” should be corrected to “with the expected”.

3. The description of root developmental stages is inconsistent between the Results and Methods sections. In Lines 242–253, the R2 stage is described as “callus proliferation” and the R3 stage as “primary root tips appearance”, whereas in Lines 517-521, R2 is described as “root primordium initiation” and R3 as “root differentiation”. Please harmonize the definitions of all developmental stages throughout the manuscript.

4. In Fig 4 and Fig 6, the heatmaps and UMAP labels are difficult to read due to the small font size. Please ensure that all figure labels, legends, and gene names meet the journal’s publication standards for readability.

**Response to Minor points:** Thank you for these careful suggestions. We have carefully checked the manuscript and revised the corresponding issues as follows.

- We have checked all the gene names throughout the manuscript, Gene names are now consistently italicized, whereas protein names are presented in non-italics.
- We have carefully proofread the manuscript and corrected typographical errors.
- We sincerely apologize for the inconsistency description of root developmental stages in the original manuscript. We have revised the stage definitions throughout the Results and Methods sections. Specifically, R2 is now consistently defined as the stage of callus proliferation and root primordium initiation, while R3 is defined as the stage of root differentiation and adventitious root tip appearance. We also replaced “primary root tips” with “adventitious root tips” to more accurately describe the tissue-culture-induced rooting system.
- The font size of Fig. 4 and Fig. 6 has been revised to improve readability and meet the journal’s publication standards.

**Reviewer #3:**

This study presents a single-cell transcriptomic atlas of leaf, stem, and root tissues in Quercus robur and identifies QrIAA14-1 as a candidate regulator associated with root development. The generation of a multi-organ single-cell resource in oak is technically valuable, particularly given the challenges associated with protoplast isolation from woody tissues. The dataset will likely provide a useful community resource for future studies of woody plant development.

However, although the dataset itself is potentially valuable, I think that the current manuscript substantially overstates the mechanistic significance of the findings. In particular, the central conclusion that “IAA14 is a critical repressor of adventitious root formation” is not sufficiently supported by the presented evidence. At present, the study mainly provides correlative evidence based on expression patterns and overexpression phenotypes, without sufficiently demonstrating causality or mechanism.

Overall, I consider the manuscript interesting but not yet suitable for publication in its current form. Major revisions would be required to support the major claims.

**Major comments**

1. One important strength of this study is the successful generation of scRNA-seq datasets from multiple vegetative organs of a woody species, particularly stem tissues, which are technically challenging due to lignification, rigid tissue structure, and difficulties in protoplast isolation.
However, the manuscript currently does not sufficiently discuss the technical challenges associated with generating these datasets or how these issues may have influenced cell recovery and dataset composition. Additional discussion regarding:

• optimization of protoplast isolation,

• recovery efficiency of vascular/lignified cell populations,

• potential dissociation and digestion biases,

• and limitations in cell-type representation would substantially strengthen the manuscript.

For example, mature lignified vascular cells may be underrepresented in stem datasets, whereas leaf tissues may preferentially yield highly viable mesophyll and epidermal populations. Similarly, some differentiated root vascular populations may be more difficult to recover than meristematic cell types.

In addition, the Discussion should more explicitly address the potential influence of protoplasting-induced stress responses on transcriptional states and how such effects may differ among organs.

Given the technical difficulty of obtaining high-quality scRNA-seq datasets from woody tissues, a more balanced discussion of both the strengths and limitations of the dataset would help readers better interpret the biological conclusions and appreciate the technical value and scope of this resource.

**Response:** Thank you for this helpful suggestion. We agree that protoplast isolation is a critical step for plant scRNA-seq, especially in woody species. We have revised the Discussion to address key technical considerations associated with protoplast dissociation in woody tissues, including digestion efficiency, lignified cell recovery, potential dissociation bias, and protoplasting-induced transcriptional perturbations. We also suggested that scRNA-seq findings are more informative when evaluated together with complementary molecular, physiological, and functional evidence. In addition, we have added more details related to the optimized protoplast isolation strategy in the Materials and Methods section, including enzyme composition, digestion time, filtration, washing, and cell viability assessment. These revisions provided a more balanced discussion of both the strengths and limitations of the scRNA-seq dataset generated in this study. The details are provided in the revised Materials and Methods and Discussion section, **Lines 516-524** and **Lines 376-395**, respectively.

2. The mechanistic evidence supporting QrIAA14-1 as a “critical regulator” of adventitious root formation remains insufficient.

The central conclusion of the manuscript is that QrIAA14-1 functions as a key negative regulator of root development in oak. However, the current evidence is primarily based on gene expression analyses and overexpression phenotypes. While these results suggest a potential association between QrIAA14-1 and root development, they are not sufficient to establish a mechanistic or endogenous regulatory role.

This limitation is particularly important for AUX/IAA family members, which are well known to exhibit functional redundancy, dosage sensitivity, and strong context-dependent effects in auxin signaling pathways.

At present, the data mainly demonstrate that overexpression of QrIAA14-1 inhibits root growth. However, such phenotypes are not unexpected for AUX/IAA-related regulators and do not by themselves establish that QrIAA14-1 is a critical endogenous regulator of adventitious rooting in oak.

Therefore, the authors should either substantially moderate their conclusions or provide stronger functional evidence supporting the proposed regulatory role of QrIAA14-1.

**Response:** Thank you for this important and constructive comment. We have carefully revised the Title, Abstract, Results, Discussion, and Conclusion to substantially improve the manuscript.

To strengthen the functional evidence, we added an additional independent *QrIAA14-1* overexpression line and quantified both adventitious root length and root number. The revised results showed that *QrIAA14-1* overexpression significantly reduced adventitious root length, whereas adventitious root number was not significantly different from the control, suggesting that *QrIAA14-1* mainly affects adventitious root elongation rather than completely blocking root initiation. In addition, we performed a co-expression analysis between the four candidate *QrIAA* genes and *QrARF* family members. Notably, *QrIAA14-1* showed the highest weighted degree in the QrIAA-QrARF co-expression network, and identified seven QrIAA14-1-associated *QrARF* candidates. Among which, *QrARF8-1* displayed the prominent expression along the epidermis-to-root hair developmental trajectory. These results provide additional support for a putative QrIAA14-1-associated auxin regulatory module. The corresponding revisions and new analyses are provided in **Fig. 6e-f**, **Fig. S4**, and **Tables S10-S12**, and relevant revisions are described in **Lines 319-330**, **Lines 479-487**, **Lines 605-612**, **Lines 679-687**, and **Lines 820-824** of the revised manuscript.

3. The reproducibility and variability of the QrIAA14-1 overexpression phenotype are currently difficult to evaluate.

Although the Methods state that at least five independent transgenic lines were analyzed, neither the main figures nor the supplementary figures present line-specific expression levels or phenotypic measurements. Because overexpression lines are expected to vary substantially due to insertion-site and copy-number effects, it is important to show the range of transgene expression levels and associated root phenotypes across independent lines.

The authors should provide line-specific data including QrIAA14-1 expression levels and root phenotypes for each independent line, the meaning of the error bars, and details of the statistical analyses.

**Response**: Thank you for this important and constructive suggestion. We agree that line-specific expression and phenotypic data are important for evaluating the reproducibility and variability of the *QrIAA14-1* overexpression phenotype. We apologize for the inaccurate description in the previous Materials and Methods section, and we have corrected this statement and clarified that two independent transgenic lines were investigated to explore the gene function, and four plantlets of each line were analyzed for phenotypic evaluation.

To address this concern, we added an additional independent overexpression line and performed line-specific analyses of *QrIAA14-1* expression levels, adventitious root length, and adventitious root number in the revised manuscript (**Fig. 6e-f**). The results showed that both independent *QrIAA14-1* overexpression lines exhibited significantly reduced adventitious root length compared with the *35S:EGFP* control, whereas the number of adventitious roots was not significantly different from that of the control. These additional data support a more precise conclusion that *QrIAA14-1* mainly affects adventitious root development and elongation, rather than strictly preventing adventitious root initiation. The corresponding Results, Materials and Methods, and Legend of Fig. 6 have been revised in the **Lines 349-353**, **Lines 664-665** and **Lines 820-824** of the revised manuscript, respectively.

4. The classification of QrIAA14-1 and other AUX/IAA proteins as “transcription factors” throughout the manuscript should be reconsidered.

AUX/IAA proteins are generally regarded as auxin-responsive transcriptional repressors that function through interactions with ARF transcription factors, rather than canonical DNA-binding transcription factors themselves.

Therefore, referring to QrIAA14-1 as a “key transcription factor” may not be mechanistically accurate and could create conceptual confusion regarding its molecular role within the auxin signaling pathway.

The terminology should be revised throughout the manuscript to more clearly distinguish ARF transcription factors from AUX/IAA transcriptional repressors.

**Response:** Thank you for this important comment. We agree that AUX/IAA proteins, including *QrIAA14-1*, should not be described as canonical DNA-binding transcription factors. We have carefully checked the manuscript and revised the terminology throughout the text to clearly distinguish ARF transcription factors from AUX/IAA transcriptional repressors. Specifically, descriptions of AUX/IAA as “transcription factors” were revised to “transcriptional regulators” or “transcriptional repressors” in the revised manuscript.

5. The interpretation of QrIAA14-1 expression patterns requires additional clarification and more cautious terminology.

The manuscript states that IAA14-1 and IAA14-2 were “mainly expressed in root hair and near root hair cells, with no detectable signals in the epidermal layer.” However, root hair cells themselves are specialized epidermal cell types, making this distinction conceptually unclear.

In addition, the presented expression patterns in UMAP appear broader than described, with detectable expression also observed in additional cell populations, including vascular-associated clusters. Therefore, the current wording may overstate the degree of cell-type specificity.

The authors should more carefully distinguish between preferential expression and true cell-type specificity. In particular, root hair cells should be discussed as specialized epidermal lineage cells rather than as completely separate cell types from the epidermis.

More cautious interpretation of the expression patterns and cell annotations would improve the biological clarity and rigor of the manuscript.

**Response:** Thank you for this helpful comment. We sincerely apologize for the imprecise wording in the previous version, which may have implied a strict separation between root hair cells and epidermal cells. In fact, our intention was to describe the expression pattern of *QrIAA14-1* and *QrIAA14-2* along the epidermis-to-root hair developmental trajectory, where these genes showed higher expression toward near-root-hair and root hair cell states. We have revised the Results and Discussion section to clarify that root hair cells are specialized epidermal-lineage cells. The details are provided in **Lines 336-339** and **Lines 459-463** of the revised manuscript.

6. The RNA in situ hybridization results presented in Fig. 6d are not fully convincing and require clearer validation and interpretation.

The nature of the “Mock” control used in the RNA in situ hybridization experiments should be clarified more explicitly.

In general, comparison between antisense and corresponding sense probes is important for evaluating signal specificity in RNA in situ hybridization experiments. However, it is currently unclear whether the “Mock” control represents a sense probe control, a no-probe control, or another experimental condition.

Because the signal-to-background contrast in Fig. 6d appears relatively subtle, clear definition of the negative control is particularly important for interpreting the specificity of the observed staining patterns.

In addition, the spatial localization observed in the *in situ* images does not appear fully consistent with the scRNA-seq-based interpretation.

**Response**: Thank you for this helpful suggestion. We have revised the Materials and Methods section, Results, and **Fig. 6d** to clarify the information regarding sense and antisense probes in the RNA *in situ* hybridization assay. Specifically, the SP6-transcribed sense probe was used as the negative control, whereas the T7-transcribed antisense probe was used to detect QrIAA14-1 transcripts. Compared with the SP6 sense-probe control, positive *QrIAA14-1* hybridization signals were detected in the T7 antisense-probe samples, particularly in the root primordium-associated tissues at the R2 stage and in the root tip (especially root cap) region at the R5 stage.

Moreover, the UMAP analysis showed that *QrIAA14-1* was significantly enriched in root-tip/root cap-associated populations, with detectable expression also present in additional root cell populations, including vascular-associated clusters. In addition, the root hair cells are small and difficult to clearly preserve in paraffin sections, so the RNA *in situ* hybridization mainly validated the spatial enrichment of *QrIAA14-1* in root primordium-associated tissues and the root tip/root cap region. Instead, the *in situ* hybridization results mainly support the spatial enrichment of *QrIAA14-1* in root primordium-associated tissues and the root tip/root cap region, which is consistent with the scRNA-seq-based significant enrichment of *QrIAA14-1* in root cap-associated populations (Fig. 4i). The details are provided in **Lines 346-349**, **Lines 625-630** and **Lines 815-819** of the revised manuscript.

7. The terminology regarding root identity and developmental stage requires clarification throughout the manuscript.

The experimental system appears to involve de novo root regeneration from stem explants during tissue culture propagation, which would generally be classified as adventitious root formation rather than primary root development. However, the manuscript refers to “primary root” in the abstract and main text.

Because primary roots and adventitious roots arise through distinct developmental programs, the current terminology creates conceptual ambiguity regarding the biological process being analyzed. More precise terminology throughout the manuscript would substantially improve clarity.

**Response:** Thank you for this important comment. We have carefully checked the entire manuscript and revised the terminology related to root identity and developmental stages. Specifically, “primary root” and related descriptions were replaced with “adventitious root”, “adventitious root tips”, or “adventitious root development” in the revised manuscript, including the Abstract, Materials and Methods, Results, Discussion, Conclusion, and figure legends. These revisions improve the clarity and accuracy of the biological process investigated in this study.

8. p13, lines 341–346:

The statements in this section appear somewhat overstated relative to the analyses presented in the manuscript.

Although the study successfully generates scRNA-seq datasets from multiple vegetative organs, the analyses are largely performed separately for each organ. Therefore, it is unclear whether the manuscript truly enables a “systematic comparison” of developmental trajectories and regulatory programs across organs, as currently stated.

Similarly, the phrase “dissecting the organ-specific regulatory mechanisms” may overstate the mechanistic depth of the study, which primarily identifies candidate genes and transcriptional patterns rather than directly resolving regulatory mechanisms.

More cautious wording would improve the accuracy and balance of the Discussion.

**Response:** Thank you for this helpful comment. We have revised the Title and Discussion to use more cautious wording. Specifically, we now describe the study as providing an organ-resolved view of cellular composition and transcriptional dynamics across leaf, stem, root tissue, and as identifying organ-enriched cell populations and candidate regulatory genes, thereby establishing a valuable working framework for future mechanistic studies of vegetative organ development in oaks. The details are provided in **Lines 371-374** of the revised manuscript.

9. p15, lines 415-418

The interpretation of the temporal relationship between QrIAA14-1 expression and IAA accumulation appears somewhat speculative.

While the data show that QrIAA14-1 expression peaks earlier than the maximum IAA accumulation, the proposed model suggesting that “early IAA14-1 induction may desensitize auxin signaling to prevent premature differentiation” is not directly supported by the presented experiments.

In particular, the manuscript does not include direct analyses of auxin signaling activity, signaling sensitivity, or differentiation status. Therefore, the current data support a temporal association rather than a mechanistic regulatory cascade.

More cautious interpretation of these observations would

**Response:** Thank you for this important and constructive comment. We have rewritten the relevant section to improve the Discussion. Specifically, we revised that *QrIAA14-1* expression peaked at the R3 stage, whereas endogenous auxin reached its maximum at the R5 stage, suggesting a temporal association that may reflect a feedback-associated regulatory response within the auxin signaling pathway. We also added a future perspective that auxin-responsive reporters, *ARF* target-gene analyses, and auxin sensitivity assays could further clarify the potential mechanism by which *QrIAA14-1* responds to auxin and regulates oak adventitious root development. The details are provided in **Lines 465-479** of the revised manuscript.

10. The current title and overall framing of the manuscript may somewhat overstate both the analytical scope and the mechanistic depth of the study.

In particular, the phrase “single-cell multi-omics integration” suggests a more extensive integrative analytical framework than is currently presented. Although the manuscript combines scRNA-seq, gene expression analyses, and hormone measurements, these datasets are largely analyzed separately rather than through a unified integrative framework.

Similarly, the title places substantial emphasis on QrIAA14-1 as a central mechanistic regulator of adventitious root formation. However, the functional evidence currently presented for QrIAA14-1 remains relatively limited and is primarily based on expression analyses and overexpression phenotypes.

In contrast, one of the major strengths of the study is the generation of a multi-organ scRNA-seq resource for a woody species. The manuscript may therefore be stronger if framed more cautiously as a resource and candidate-gene study rather than as a mechanistic characterization centered on QrIAA14-1.

More balanced wording in the title and Abstract would better reflect the scope and strengths of the study.

**Response:** Thank you for this important and constructive suggestion. We agree that the previous title and framing may have overstated the mechanistic depth of the study. Therefore, we have revised the title and the relevant descriptions in the Abstract, Results, Discussion, and Conclusion to more accurately frame the study as a multi-organ single-cell transcriptomic resource combined with candidate-gene identification and validation in the revised manuscript.

**Minor Points:**

1. p6, line80 the major cell types of tissues, such as cotyledons, leaves, stems and roots. “Cotyledons”, “leaves”, “stems”, and “roots” are organs rather than tissues. The terminology should therefore be revised for accuracy.

2. p9, line 166: “the genes in the C2 cluster within the C2 cluster displayed sustained transcriptional activity”

The phrase “in the C2 cluster within the C2 cluster” is redundant and should be corrected.

3. p11, line 242: “The root development of Q. robur plantlets was divided into five distinct stages”. The terminology here appears overly general. The described process involves de novo root regeneration/rooting from stem explants during tissue culture propagation rather than general root development. More precise terminology (e.g., “adventitious root formation” or “root regeneration process”) would improve biological clarity.

4. p13, line336 ScRNA -> scRNA

5. Several citations throughout the manuscript are missing spaces before the reference brackets (e.g., “differentiation [51]” instead of “differentiation [51]”). Please revise the formatting consistently throughout the manuscript.

6. p17, line511: The phrase “were considered as significantly differentially expression” is grammatically incorrect and should be revised to “were considered significantly differentially expressed”.

**Response to Minor Points:** Thank you for these careful suggestions. We have carefully checked the manuscript and revised the corresponding issues as follows.

- We have revised the terminology by replacing “tissues” with “organs” when referring to cotyledons, leaves, stems, and roots.
- We have corrected the redundant phrase “in the C2 cluster within the C2 cluster” to “in the C2 cluster” in the revised manuscript.
- We have revised the terminology related to root developmental stages. Specifically, the general term “root” was replaced with “adventitious root” in the revised manuscript.
- We have corrected “ScRNA” to “The scRNA” in the Line 359 of revised manuscript.
- We have carefully checked the reference formatting throughout the manuscript and added missing spaces before reference brackets in the revised manuscript.
- We have proofread the entire manuscript to correct the grammatical error.
