# Supplementary material for: Multi-organ single-cell transcriptomic atlas identifies QrIAA14 as a candidate negative regulator of adventitious root development in Quercus robur
Source: PLoS Genet. 2026 Sep 1;22(9):e1012300. doi: 10.1371/journal.pgen.1012300 (PMC13552951; doi:10.1371/journal.pgen.1012300)
Supplement: Supplementary file 7 [file pgen.1012300.s018.docx]

They conducted single-cell RNA-seq in the leaves, stems, and roots of *Quercus robur* to identify the differences among the three organs. In addition, they conducted bulk RNA-seq at different stages of adventitious root regeneration, from callus on the stem, to explore the suppressive factors of root development in *Q. robur*. They also conducted phytohormone analysis, *in situ* hybridization, and morphological analysis using an overexpression line to confirm their sequence data. The extensive experiments provide a valuable dataset and suggest that *QrIAA14-1* repressed adventitious root initiation and development in *Q. robur*. While the study provides a wide dataset on woody plants, especially oak, there are several areas where the material and methods section, data presentation, and the strength of the conclusions need clarification and improvement.

Major points

1. The use of the word "critical" in the title is too strong to describe *QrIAA14-1* as a repressor of adventitious root formation. The authors described that *QrIAA14-1* suppresses root initiation and development in the manuscript, but roots are still induced in the *QrIAA14-1* overexpression line. This data suggests that *QrIAA14-1* may play a partial role in suppressing root development rather than strictly repressing root initiation. I recommend changing the title and some parts of the manuscript. It would also be helpful to show a time-course analysis of adventitious root numbers and lengths in the wild-type and *QrIAA14-1* overexpression lines, or to provide data on the number of adventitious roots in a *Qriaa14-1* loss-of-function mutant, which would support the conclusion.
2. In line 246, the authors described that primary root tips appeared in R3 stage. However, it is difficult to recognize the roots or callus in R3 stage from the current images (Fig 5a). Please provide higher-resolution images or a magnified image for R3 stage in Fig. 5a.
3. The Materials and Methods section lacks sufficient explanation; please clarify the methods to ensure reproducibility.

- About materials: Please specify the source or origin of the *Q. robur* plants. For the "4-week-old" plants (line 458), clarify whether this is from seed germination or shoot meristem culture. Additionally, describe the growth conditions (soil or culture media, type of growth chamber).
- Please provide the source of the reference genome (line 473).
- Please describe the methods of RNA isolation and cDNA reaction in RT-qPCR (line 544).
- Please describe the methods of phytohormone analysis: extraction and purification protocols, and specify the company of the instrument. Importantly, clarify which exact plant parts (whole plants, specific tissues, callus, or root parts) were collected for the 100 mg samples.

4. The developmental states of the plants used for each stage appear inconsistent in Fig. 5a. For instance, R0 plants lack internodes, whereas others have multiple internodes. Leaves are cut in half in R0–R3, but R4 and R5 differ. The R2 callus appears larger than that in R3, and leaf numbers vary. If whole plants were sampled for bulk RNA-seq, RT-qPCR, and phytohormone analyses, these morphological inconsistencies would affect the results. Please clarify which tissues or whole plants were sampled and describe how these sample variations were controlled.
5. Interpreting the results of *in situ* hybridization (Fig. 6d) was difficult.

- The labels "mock" and "*QrIAA14-1*" should be corrected to standard terminology, such as "sense" and "anti-sense" probes.
- The signals in both the R2 and R5 stages are difficult to distinguish. In addition, please explain what the red arrows indicate.
- Please provide a diagram or describe the exact sectioning plane or position used for the R2 stage, as it is currently difficult to identify the tissue part.

6. In Fig 6d, the differences between the *p35S:GFP* and *p35S: QrIAA14-1:GFP* signals are not visually recognizable. The high background signal and prominent artifacts complicate interpretation, and there are concerns about the possibility of overexposure. Furthermore, the imaging conditions (e.g., microscope and exposure time) are not provided in the Materials and Methods. Please clarify the exact purpose of this image comparison in the text and provide cleaner images with appropriately optimized exposure settings. Additionally, please include imaging parameters in the Methods section.
7. Lines 230–231 mentioned that conducting KEGG enrichment analysis focuses on the root cap and near root hair cells. Since the study focuses on adventitious root initiation and development, focusing on the root meristem would seem more appropriate. Please provide a scientific rationale for choosing the root cap and root hair cells over the meristematic zone.
8. There are inconsistencies in the numerical data between the main text and the supplementary tables (e.g., line 115 mentions 6,892 cells, but the S2 table mentions 5,943 cells. Line 251: 44.17% as well). Please carefully review and correct all numerical data for consistency.
9. The statement “The leaf-derived cells were predominantly distributed in 17 clusters (Fig. 1c), with cluster 2, cluster 25, and cluster 27 consisting of more than 98%.” (lines 121–122) is unclear. Please clarify the denominator for this 98% and how this number was calculated.

Minor points

1. Please describe the company names for all key chemicals, kits, and equipment mentioned in the Methods.
2. Provide the full name of a gene only at its first mention in the text (e.g., lines 55-56, lines 529-530). Thereafter, consistently use the abbreviation.
3. Both "plant hormones" and "phytohormones" are used in the manuscript. Please choose one term and use it consistently throughout.
4. There are extra closing parenthese and a period ")." at line 121.
5. Please arrange the cluster numbers for each organ in Table S3 in numerical order to improve readability.
