# Supplementary material for: Multi-organ single-cell transcriptomic atlas identifies QrIAA14 as a candidate negative regulator of adventitious root development in Quercus robur
Source: PLoS Genet. 2026 Sep 1;22(9):e1012300. doi: 10.1371/journal.pgen.1012300 (PMC13552951; doi:10.1371/journal.pgen.1012300)
Supplement: Supplementary file 8 [file pgen.1012300.s021.docx]

***Point-by-point response***

**Reviewers' comments:**

**Reviewer #1:** The authors have adequately improved their manuscripts and addressed the majority of the points raised in the previous review. I have some minor comments remaining.

1. Methods of phytohormone quantification (from line 647 to 653, and Fig. 5g)

・Please clarify how the samples were collected, specifically, whether they were freeze-dried or simply frozen during sampling. In addition, please describe how the various phytohormones were extracted and purified.

・Which internal standards were used for phytohormone quantification, and in what volume or concentration were they added?

・In lines 651-653 and Fig. 5g: “indole-3-acetic acid (auxin compound)” should be written as “indole-3-acetic acid (IAA)” because "AUX" typically refers to genes or proteins rather than the hormone itself. Similarly, the prefix "trans" in "trans-zeatin" should be italicized and "TZ" should be corrected to "tZ", and "TZR" to "tZR", following standard nomenclature. Additionally, please clarify whether jasmonic acid or jasmonic acid-isoleucine (JA-Ile) was analyzed.

・The vertical axis label in Fig. 5g, "concentration (ng/g)" should be revised to specify the units more precisely, e.g., "concentration (ng/g fresh weight)" or "concentration (ng/g dry weight)," depending on how the samples were processed.

**Response:** Thank you for these helpful and detailed suggestions. We have revised the Materials and Methods section, Results section, Fig. 5g, and the corresponding Figure Legend to clarify the phytohormone quantification procedure and the nomenclature.

- We have clarified the sampling procedure of the phytohormones extraction and purification. The details are provided in **Lines 667-674** of the revised manuscript.
- The phytohormone quantification contained isotope-labeled internal standards, including D5-IAA, D6-ABA, D2-GA4, D5-JA, D5-tZ, D5-tZR, D4-SA, and D4-ACC, each at a final concentration of 2 ng/mL. The details are provided in **Lines 669-671** of the revised manuscript.
- We have corrected the phytohormone nomenclature throughout the manuscript according to your suggestion. Additionally, we clarified that jasmonic acid (JA), rather than jasmonic acid-isoleucine (JA-Ile), was quantified in this study. The details are provided in **Lines 661-665** of the revised manuscript.
- Approximately 100 mg of fresh sample was collected for each sample, immediately frozen in liquid nitrogen, and stored at -80°C until extraction. The samples were not freeze-dried before phytohormone extraction. Therefore, the vertical axis in Fig. 5g has been revised to “Concentration (ng/g FW)”. The details are provided in **Lines 664-665** and **Lines 843-845** of the revised manuscript.

2. Were the cell walls also stained in the sections of *in situ* hybridization shown in Fig. 6d? If so, please clarify the staining method used.

**Response:** Thank you for this helpful comment. We have clarified the staining and signal-detection procedures used for the RNA *in situ* hybridization assays.

The sections were lightly counterstained with 0.1% eosin in 100% ethanol before paraffin embedding and sectioning, which helped visualize cell walls and cellular structures during tissue preparation. No additional cell-wall-specific staining was performed after hybridization. The *QrIAA14-1* hybridization signals were detected using the DIG-labeled probe system with anti-digoxigenin alkaline phosphatase and visualized with NBT/BCIP chromogenic substrate.

The corresponding **Materials and Methods section** have been provided in **Lines 647-656** of the revised manuscript.

3. Although red arrowheads have been added in Fig. 6d and described in the manuscript, it is unclear from the figure whether they indicate a specific region or a small signal in R5 stage. Since arrowheads typically indicate a specific point rather than an area, if the intention is to highlight a region, it would be clearer to use a different symbol (e.g., a bracket) instead of arrowheads.

**Response**: Thank you for this helpful suggestion. To avoid this confusion, we have removed the red arrowheads from Fig. 6d, and revised the figure legend to clarify the interpretation of the RNA in situ hybridization results.

Specifically, *QrIAA14-1* hybridization signals were dominantly detected in the root cap region of the T7 antisense-probe samples at the R5 stage, whereas weaker staining was observed in other root regions as well as in the SP6 sense-probe control.

The corresponding revisions have been made in **Fig. 6d** and the **Figure Legend of Fig. 6d** in the revised manuscript.

4. In Fig. 6e, the image for "35S:QrIAA14-1.2" is out of focus, so please replace it with a higher-quality image if possible.

**Response:** Thank you for these helpful and detailed suggestions. We have replaced the image of "35S:QrIAA14-1.2" in **Fig. 6e**.

5. S5 Table, cell A36 contains the value "10," which appears inconsistent with the surrounding data. Please confirm whether this value is correct. Additionally, the column headers appear to be missing in S5 Table, so please add them for clarity.

**Response:** Thank you for your careful checking, and we apologize for this oversight. We have carefully re-checked and corrected S5 Table, and further reviewed the entire table to ensure data consistency. In addition, we have added the missing column headers to S5 Table to improve clarity and readability. The revised **S5 Table** has been updated accordingly.

**Reviewer #2:** The authors have made substantial revisions to the manuscript. All previous concerns regarding methodology and data presentation have been adequately addressed. I am satisfied with the quality of the revisions and recommend acceptance.

**Response:** Thank you very much for your positive evaluation of our revised manuscript. We sincerely appreciate your careful review and constructive comments, which helped us improve the quality and clarity of the manuscript.

**Reviewer #3:** The authors have adequately addressed my previous comments. I have one minor comment regarding the newly added QrIAA-QrARF co-expression analysis.

ARF proteins can function as either transcriptional activators or repressors, and the newly identified QrIAA14-1-associated candidates appear to include members potentially belonging to both classes. This distinction is important for interpreting the proposed QrIAA14-1-ARF regulatory module, because an interaction with an activator-type ARF would have a different mechanistic implication from an association with a repressor-type ARF.

The authors should therefore indicate whether the identified QrARFs, particularly QrARF8-1, are predicted to be activator- or repressor-type ARFs, preferably based on phylogenetic relationships and/or the amino-acid composition of their middle regions. A brief discussion of how this classification relates to the proposed inhibitory effect of QrIAA14-1 on adventitious root elongation would improve the mechanistic interpretation of the newly added analysis. Because these functions have not been experimentally demonstrated in Q. robur, the classification should be presented as putative.

**Response:** Thank you for this insightful and constructive comment. We agree that distinguishing activator- and repressor-type ARFs is important for our findings and further study.

ARF proteins can be broadly classified into activator- and repressor-type members, and this functional distinction is largely associated with their phylogenetic grouping and the amino-acid composition of the middle region. Previous studies shown that *ARF8-like* genes are positive regulators of early adventitious root formation and root developmental responses in *Arabidopsis* and *Populus*. Consistently, *QrARF8-1* showed prominent expression along the epidermis-to-root-hair developmental trajectory in oak roots, suggesting it may be involved in auxin-associated root cell differentiation. Thus, future studies could employ the *QrIAA14-1* loss-of-function materials, ARF-AUX/IAA interaction assays, and auxin-responsive reporter analyses to clarify the underlying roles of *QrARF8-1*-*QrIAA14-1* module in responding to auxin signaling and regulating oak root development, which will provide a potential mechanistic insight into the hormone-centered regulatory framework of adventitious root regeneration in woody species.

The corresponding **Discussion section** have been provided in **Lines 498-509** of the revised manuscript.
