# Supplementary material for: Multi-organ single-cell transcriptomic atlas identifies QrIAA14 as a candidate negative regulator of adventitious root development in Quercus robur
Source: PLoS Genet. 2026 Sep 1;22(9):e1012300. doi: 10.1371/journal.pgen.1012300 (PMC13552951; doi:10.1371/journal.pgen.1012300)
Supplement: Peer review history

# Peer review: Decision Letter 0

**DOI:** 10.1371/journal.pgen.1012300.r001

**Authors:** Angela Hancock, Tomokazu Kawashima

22 May 2026

PGENETICS-D-26-00346

Single-cell multi-omics integration reveals IAA14 as a critical repressor of adventitious root formation in Quercus robur

PLOS Genetics

Dear Dr. Hui,

Thank you for submitting your manuscript to PLOS Genetics. After careful consideration, we feel that it has merit but does not fully meet PLOS Genetics's publication criteria as it currently stands. Therefore, we invite you to submit a revised version of the manuscript that addresses the points raised during the review process.

Please submit your revised manuscript by Jul 21 2026 11:59PM. If you will need more time than this to complete your revisions, please reply to this message or contact the journal office at plosgenetics@plos.org. When you're ready to submit your revision, log on to https://www.editorialmanager.com/pgenetics/ and select the 'Submissions Needing Revision' folder to locate your manuscript file.

Please include the following items when submitting your revised manuscript:

* A letter that responds to each point raised by the editor and reviewer(s). You should upload this letter as a separate file labeled 'Response to Reviewers'. This file does not need to include responses to any formatting updates and technical items listed in the 'Journal Requirements' section below.

* A marked-up copy of your manuscript that highlights changes made to the original version. You should upload this as a separate file labeled 'Revised Manuscript with Track Changes'.

* An unmarked version of your revised paper without tracked changes. You should upload this as a separate file labeled 'Manuscript'.

If you would like to make changes to your financial disclosure, competing interests statement, or data availability statement, please make these updates within the submission form at the time of resubmission. Guidelines for resubmitting your figure files are available below the reviewer comments at the end of this letter.

As the corresponding author, your ORCID iD is verified in the submission system and will appear in the published article. PLOS supports the use of ORCID, and we encourage all coauthors to register for an ORCID iD and use it as well. Please encourage your coauthors to verify their ORCID iD within the submission system before final acceptance, as unverified ORCID iDs will not appear in the published article. Only the individual author can complete the verification step; PLOS staff cannot verify ORCID iDs on behalf of authors.

We look forward to receiving your revised manuscript.

Kind regards,

Tomo Kawashima

Academic Editor

PLOS Genetics

Angela Hancock

Section Editor

PLOS Genetics

Aimée Dudley

Editor-in-Chief

PLOS Genetics

Anne Goriely

Editor-in-Chief

PLOS Genetics

**Journal Requirements:**

1) Please ensure that the CRediT author contributions listed for every co-author are completed accurately and in full.

At this stage, the following Authors/Authors require contributions: Wenkai Hui, Jiayue Li, Hao Li, Hongyi Wu, Yi He, Shuangying Yang, Xiong Huang, Hanbo Yang, Gang Chen, Peng Zhu, Xiaohong Chen, Xingcui Xiao, Yu Zhong, Zhenfeng Xu, and Fang He. Please ensure that the full contributions of each author are acknowledged in the "Add/Edit/Remove Authors" section of our submission form.

The list of CRediT author contributions may be found here: https://journals.plos.org/plosgenetics/s/authorship#loc-author-contributions

2) Please provide an Author Summary. This should appear in your manuscript between the Abstract (if applicable) and the Introduction, and should be 150-200 words long. The aim should be to make your findings accessible to a wide audience that includes both scientists and non-scientists. Sample summaries can be found on our website under Submission Guidelines:

https://journals.plos.org/plosgenetics/s/submission-guidelines#loc-parts-of-a-submission

3) We do not publish any copyright or trademark symbols that usually accompany proprietary names, eg ©,  ®, or TM  (e.g. next to drug or reagent names). Therefore please remove all instances of trademark/copyright symbols throughout the text, including:

- TM on page: 14.

4) Please upload all main figures as separate Figure files in .tif or .eps format. For more information about how to convert and format your figure files please see our guidelines:

https://journals.plos.org/plosgenetics/s/figures

5) Some material included in your submission may be copyrighted. According to PLOS's copyright policy, authors who use figures or other material (e.g., graphics, clipart, maps) from another author or copyright holder must demonstrate or obtain permission to publish this material under the Creative Commons Attribution 4.0 International (CC BY 4.0) License used by PLOS journals. Please closely review the details of PLOS's copyright requirements here: <a data-cke-saved-href="https://journals.plos.org/plosgenetics/s/licenses-and-copyright" href="https://journals.plos.org/plosgenetics/s/licenses-and-copyright">PLOS Licenses and Copyright</a>. If you need to request permissions from a copyright holder, you may use the&nbsp;<a data-cke-saved-href="https://storage.googleapis.com/plos-published-prod/c4ff/Content%20Copyright%20Permission%20Form.pdf?X-Goog-Algorithm=GOOG4-RSA-SHA256&amp;X-Goog-Credential=wombat-sa%40plos-prod.iam.gserviceaccount.com%2F20220817%2Fauto%2Fstorage%2Fgoog4_request&amp;X-Goog-Date=20220817T210608Z&amp;X-Goog-Expires=86400&amp;X-Goog-SignedHeaders=host&amp;X-Goog-Signature=cd9dc868773378f7033b9f8cfb91430167d6275df90bce01342a454f36512530b64f24736fbf68c4fd96da74d663b6ee95b3420bded8fb0e833af2390b896b8706643b476d7f532408921b60576d65b60617070900fd2bcd28eec4cd32fefcf007d3655df17fcc91023329a28479ce0d1c5758174eedcc9e3c1788c5f7dd8523f3ddd871e02c998a20f7dc78ec00f313d4b5a78ba2037a6c7ef0a05dc29817da9368065460c8d8446659f35f0458fefabd3101eb7f926e9cb7a0b53e7bbe47aaed3e0ba52f9af9c2b8f6d7d3a8871b205fc4cbe97384ed37c7604fd7eecfedccf49cba12820b2e3b649541cefde3103cd7d72731b3e8b0248439b4cba691ca8a" href="https://storage.googleapis.com/plos-published-prod/c4ff/Content%20Copyright%20Permission%20Form.pdf?X-Goog-Algorithm=GOOG4-RSA-SHA256&amp;X-Goog-Credential=wombat-sa%40plos-prod.iam.gserviceaccount.com%2F20220817%2Fauto%2Fstorage%2Fgoog4_request&amp;X-Goog-Date=20220817T210608Z&amp;X-Goog-Expires=86400&amp;X-Goog-SignedHeaders=host&amp;X-Goog-Signature=cd9dc868773378f7033b9f8cfb91430167d6275df90bce01342a454f36512530b64f24736fbf68c4fd96da74d663b6ee95b3420bded8fb0e833af2390b896b8706643b476d7f532408921b60576d65b60617070900fd2bcd28eec4cd32fefcf007d3655df17fcc91023329a28479ce0d1c5758174eedcc9e3c1788c5f7dd8523f3ddd871e02c998a20f7dc78ec00f313d4b5a78ba2037a6c7ef0a05dc29817da9368065460c8d8446659f35f0458fefabd3101eb7f926e9cb7a0b53e7bbe47aaed3e0ba52f9af9c2b8f6d7d3a8871b205fc4cbe97384ed37c7604fd7eecfedccf49cba12820b2e3b649541cefde3103cd7d72731b3e8b0248439b4cba691ca8a">PLOS Content Copyright Permission&nbsp;Form</a>

Please respond directly to this email and provide any known details concerning your material's license terms and permissions required for reuse, even if you have not yet obtained copyright permissions or are unsure of your material's copyright compatibility. Once you have responded and addressed all other outstanding technical requirements, you may resubmit your manuscript within Editorial Manager.

Potential Copyright Issues:

- Please confirm (a) that you are the photographer of Figure 6E, or (b) provide written permission from the photographer to publish the photo(s) under our CC BY 4.0 license.

- Figure 1A: Please confirm whether you drew the images / clip-art within the figure panels by hand. If you did not draw the images, please provide (a) a link to the source of the images or icons and their license / terms of use; or (b) written permission from the copyright holder to publish the images or icons under our CC BY 4.0 license. Alternatively, you may replace the images with open source alternatives. See these open source resources you may use to replace images / clip-art:

- https://commons.wikimedia.org

- https://openclipart.org/.

6) Please amend your detailed Financial Disclosure statement. This is published with the article. It must therefore be completed in full sentences and contain the exact wording you wish to be published.

- State the initials, alongside each funding source, of each author to receive each grant. For example: "This work was supported by the National Institutes of Health (####### to AM; ###### to CJ) and the National Science Foundation (###### to AM).".

If you did not receive any funding for this study, please simply state: u201cThe authors received no specific funding for this work.u201d

**Reviewers' comments:**

Reviewer's Responses to Questions

**Comments to the Authors:**

**Please note here if the review is uploaded as an attachment.**

Reviewer #1: Please see the attachment.

Reviewer #2: This manuscript presents a comprehensive and high-quality single-cell transcriptomic landscape of Quercus robur. The authors have addressed the technical challenges associated with protoplast isolation in woody species and integrated scRNA-seq, bulk RNA-seq, and phytohormone profiling to identify QrIAA14-1 as a potential negative regulator of adventitious rooting. The study is timely, the methodology is sound, and the discovery of the IAA14-mediated inhibitory mechanism provides valuable insights into the vegetative propagation of forest trees. However, there still are some comments for the authors to revise the manuscript for publication.

Major Points for the Authors:

1. The authors rely heavily on Arabidopsis orthologs for cell-type annotation. I suggest providing more evidence or discussion to support whether these markers are consistently expressed in the corresponding tissues of Quercus. The authors may also consider to compare with available single-cell datasets from woody plants, such as Populus, to strengthen the reliability of cell-type annotation.

2. Although overexpression experiments have clearly demonstrated that QrIAA14-1 inhibits root elongation in oak, AUX/IAA proteins generally function by interacting with ARF transcription factors to repress auxin-responsive transcriptional activation. To determine whether a similar pathway may carry out in Q. robur, the authors should perform a co-expression analysis using the scRNA-seq or bulk RNA-seq datasets. Specifically, the authors are encouraged to identify which ARF members show coordinated expression patterns with QrIAA14-1, thereby highlighting the potential regulatory module involved in oak root development.

3. In Figure 5, the correlation between endogenous IAA content and QrIAA14-1 expression is particularly interesting. The authors should further discuss the potential feedback loop mechanism to explain how the oak tissues balance elevated auxin levels with expression of the AUX/IAA repressor during root initiation and differentiation.

4. In Discussion section, the author should further illustrate the key role of IAA14-1 in auxin signaling pathways, particularly the potential regulatory mechanisms underlying the poor rooting ability in Q. robur.

5. Please provide more details on the optimized protoplast strategy mentioned in line 380. For woody plants, the enzymatic digestion time and vacuum infiltration steps are critical for reproducibility.

Minor Points:

1. Please check the manuscript for consistent gene nomenclature, including the use of italics for gene names and non-italics for proteins.

2. Please correct minor typographical errors throughout the manuscript. For example, in Line 469, “with he expected” should be corrected to “with the expected”.

3. The description of root developmental stages is inconsistent between the Results and Methods sections. In Lines 242–253, the R2 stage is described as “callus proliferation” and the R3 stage as “primary root tips appearance”, whereas in Lines 517–521, R2 is described as “root primordium initiation” and R3 as “root differentiation”. Please harmonize the definitions of all developmental stages throughout the manuscript.

4. In Fig 4 and Fig 6, the heatmaps and UMAP labels are difficult to read due to the small font size. Please ensure that all figure labels, legends, and gene names meet the journal’s publication standards for readability.

Reviewer #3: This study presents a single-cell transcriptomic atlas of leaf, stem, and root tissues in Quercus robur and identifies QrIAA14-1 as a candidate regulator associated with root development. The generation of a multi-organ single-cell resource in oak is technically valuable, particularly given the challenges associated with protoplast isolation from woody tissues. The dataset will likely provide a useful community resource for future studies of woody plant development.

However, although the dataset itself is potentially valuable, I think that the current manuscript substantially overstates the mechanistic significance of the findings. In particular, the central conclusion that “IAA14 is a critical repressor of adventitious root formation” is not sufficiently supported by the presented evidence. At present, the study mainly provides correlative evidence based on expression patterns and overexpression phenotypes, without sufficiently demonstrating causality or mechanism.

Overall, I consider the manuscript interesting but not yet suitable for publication in its current form. Major revisions would be required to support the major claims.

＜Major comments＞

1. One important strength of this study is the successful generation of scRNA-seq datasets from multiple vegetative organs of a woody species, particularly stem tissues, which are technically challenging due to lignification, rigid tissue structure, and difficulties in protoplast isolation.

However, the manuscript currently does not sufficiently discuss the technical challenges associated with generating these datasets or how these issues may have influenced cell recovery and dataset composition. Additional discussion regarding:

• optimization of protoplast isolation,

• recovery efficiency of vascular/lignified cell populations,

• potential dissociation and digestion biases,

• and limitations in cell-type representation

would substantially strengthen the manuscript.

For example, mature lignified vascular cells may be underrepresented in stem datasets, whereas leaf tissues may preferentially yield highly viable mesophyll and epidermal populations. Similarly, some differentiated root vascular populations may be more difficult to recover than meristematic cell types.

In addition, the Discussion should more explicitly address the potential influence of protoplasting-induced stress responses on transcriptional states and how such effects may differ among organs.

Given the technical difficulty of obtaining high-quality scRNA-seq datasets from woody tissues, a more balanced discussion of both the strengths and limitations of the dataset would help readers better interpret the biological conclusions and appreciate the technical value and scope of this resource.

2. The mechanistic evidence supporting QrIAA14-1 as a “critical regulator” of adventitious root formation remains insufficient.

The central conclusion of the manuscript is that QrIAA14-1 functions as a key negative regulator of root development in oak. However, the current evidence is primarily based on gene expression analyses and overexpression phenotypes. While these results suggest a potential association between QrIAA14-1 and root development, they are not sufficient to establish a mechanistic or endogenous regulatory role.

This limitation is particularly important for AUX/IAA family members, which are well known to exhibit functional redundancy, dosage sensitivity, and strong context-dependent effects in auxin signaling pathways.

At present, the data mainly demonstrate that overexpression of QrIAA14-1 inhibits root growth. However, such phenotypes are not unexpected for AUX/IAA-related regulators and do not by themselves establish that QrIAA14-1 is a critical endogenous regulator of adventitious rooting in oak.

Therefore, the authors should either substantially moderate their conclusions or provide stronger functional evidence supporting the proposed regulatory role of QrIAA14-1.

3. The reproducibility and variability of the QrIAA14-1 overexpression phenotype are currently difficult to evaluate.

Although the Methods state that at least five independent transgenic lines were analyzed, neither the main figures nor the supplementary figures present line-specific expression levels or phenotypic measurements. Because overexpression lines are expected to vary substantially due to insertion-site and copy-number effects, it is important to show the range of transgene expression levels and associated root phenotypes across independent lines.

The authors should provide line-specific data including QrIAA14-1 expression levels and root phenotypes for each independent line, the meaning of the error bars, and details of the statistical analyses.

4. The classification of QrIAA14-1 and other AUX/IAA proteins as “transcription factors” throughout the manuscript should be reconsidered.

AUX/IAA proteins are generally regarded as auxin-responsive transcriptional repressors that function through interactions with ARF transcription factors, rather than canonical DNA-binding transcription factors themselves.

Therefore, referring to QrIAA14-1 as a “key transcription factor” may not be mechanistically accurate and could create conceptual confusion regarding its molecular role within the auxin signaling pathway.

The terminology should be revised throughout the manuscript to more clearly distinguish ARF transcription factors from AUX/IAA transcriptional repressors.

5. The interpretation of QrIAA14-1 expression patterns requires additional clarification and more cautious terminology.

The manuscript states that IAA14-1 and IAA14-2 were “mainly expressed in root hair and near root hair cells, with no detectable signals in the epidermal layer.” However, root hair cells themselves are specialized epidermal cell types, making this distinction conceptually unclear.

In addition, the presented expression patterns in UMAP appear broader than described, with detectable expression also observed in additional cell populations, including vascular-associated clusters. Therefore, the current wording may overstate the degree of cell-type specificity.

The authors should more carefully distinguish between preferential expression and true cell-type specificity. In particular, root hair cells should be discussed as specialized epidermal lineage cells rather than as completely separate cell types from the epidermis.

More cautious interpretation of the expression patterns and cell annotations would improve the biological clarity and rigor of the manuscript.

6. The RNA in situ hybridization results presented in Fig. 6d are not fully convincing and require clearer validation and interpretation.

The nature of the “Mock” control used in the RNA in situ hybridization experiments should be clarified more explicitly.

In general, comparison between antisense and corresponding sense probes is important for evaluating signal specificity in RNA in situ hybridization experiments. However, it is currently unclear whether the “Mock” control represents a sense probe control, a no-probe control, or another experimental condition.

Because the signal-to-background contrast in Fig. 6d appears relatively subtle, clear definition of the negative control is particularly important for interpreting the specificity of the observed staining patterns.

In addition, the spatial localization observed in the in situ images does not appear fully consistent with the scRNA-seq-based interpretation.

7. The terminology regarding root identity and developmental stage requires clarification throughout the manuscript.

The experimental system appears to involve de novo root regeneration from stem explants during tissue culture propagation, which would generally be classified as adventitious root formation rather than primary root development. However, the manuscript refers to “primary root” in the abstract and main text.

Because primary roots and adventitious roots arise through distinct developmental programs, the current terminology creates conceptual ambiguity regarding the biological process being analyzed. More precise terminology throughout the manuscript would substantially improve clarity.

8. p13, lines 341–346:

The statements in this section appear somewhat overstated relative to the analyses presented in the manuscript.

Although the study successfully generates scRNA-seq datasets from multiple vegetative organs, the analyses are largely performed separately for each organ. Therefore, it is unclear whether the manuscript truly enables a “systematic comparison” of developmental trajectories and regulatory programs across organs, as currently stated.

Similarly, the phrase “dissecting the organ-specific regulatory mechanisms” may overstate the mechanistic depth of the study, which primarily identifies candidate genes and transcriptional patterns rather than directly resolving regulatory mechanisms.

More cautious wording would improve the accuracy and balance of the Discussion.

9. p15, lines 415-418

The interpretation of the temporal relationship between QrIAA14-1 expression and IAA accumulation appears somewhat speculative.

While the data show that QrIAA14-1 expression peaks earlier than the maximum IAA accumulation, the proposed model suggesting that “early IAA14-1 induction may desensitize auxin signaling to prevent premature differentiation” is not directly supported by the presented experiments.

In particular, the manuscript does not include direct analyses of auxin signaling activity, signaling sensitivity, or differentiation status. Therefore, the current data support a temporal association rather than a mechanistic regulatory cascade.

More cautious interpretation of these observations would improve the rigor of the Discussion.

10. The current title and overall framing of the manuscript may somewhat overstate both the analytical scope and the mechanistic depth of the study.

In particular, the phrase “single-cell multi-omics integration” suggests a more extensive integrative analytical framework than is currently presented. Although the manuscript combines scRNA-seq, gene expression analyses, and hormone measurements, these datasets are largely analyzed separately rather than through a unified integrative framework.

Similarly, the title places substantial emphasis on QrIAA14-1 as a central mechanistic regulator of adventitious root formation. However, the functional evidence currently presented for QrIAA14-1 remains relatively limited and is primarily based on expression analyses and overexpression phenotypes.

In contrast, one of the major strengths of the study is the generation of a multi-organ scRNA-seq resource for a woody species. The manuscript may therefore be stronger if framed more cautiously as a resource and candidate-gene study rather than as a mechanistic characterization centered on QrIAA14-1.

More balanced wording in the title and Abstract would better reflect the scope and strengths of the study.

<minor comments=>

1. p6, line80 the major cell types of tissues, such as cotyledons, leaves, stems and roots

“Cotyledons”, “leaves”, “stems”, and “roots” are organs rather than tissues. The terminology should therefore be revised for accuracy.

2. p9, line 166:

“the genes in the C2 cluster within the C2 cluster displayed sustained transcriptional activity”

The phrase “in the C2 cluster within the C2 cluster” is redundant and should be corrected.

3. p11, line 242:

“The root development of Q. robur plantlets was divided into five distinct stages”

The terminology here appears overly general. The described process involves de novo root regeneration/rooting from stem explants during tissue culture propagation rather than general root development. More precise terminology (e.g., “adventitious root formation” or “root regeneration process”) would improve biological clarity.

4. p13, line336 ScRNA -> scRNA

5. Several citations throughout the manuscript are missing spaces before the reference brackets (e.g., “differentiation[51]” instead of “differentiation [51]”). Please revise the formatting consistently throughout the manuscript.

6. p17, line511

The phrase “were considered as significantly differentially expression” is grammatically incorrect and should be revised to “were considered significantly differentially expressed”.</minor>

**********

**Have all data underlying the figures and results presented in the manuscript been provided?**

Large-scale datasets should be made available via a public repository as described in the *PLOS Genetics*  data availability policy, and numerical data that underlies graphs or summary statistics should be provided in spreadsheet form as supporting information.

Reviewer #1: Yes

Reviewer #2: Yes

Reviewer #3: **No:** The manuscript states that the scRNA-seq and RNA-seq datasets are being deposited to GEO, but the accession numbers are currently unavailable.

**********

PLOS authors have the option to publish the peer review history of their article (what does this mean?). If published, this will include your full peer review and any attached files.

If you choose “no”, your identity will remain anonymous but your review may still be made public.

**Do you want your identity to be public for this peer review?**  For information about this choice, including consent withdrawal, please see our Privacy Policy.

Reviewer #1: No

Reviewer #2: No

Reviewer #3: No

[NOTE: If reviewer comments were submitted as an attachment file, they will be attached to this email and accessible via the submission site. Please log into your account, locate the manuscript record, and check for the action link "View Attachments". If this link does not appear, there are no attachment files.]

**Figure resubmission:**

While revising your submission, we strongly recommend that you use PLOS’s NAAS tool (https://ngplosjournals.pagemajik.ai/artanalysis) to test your figure files. NAAS can convert your figure files to the TIFF file type and meet basic requirements (such as print size, resolution), or provide you with a report on issues that do not meet our requirements and that NAAS cannot fix.

After uploading your figures to PLOS’s NAAS tool - https://ngplosjournals.pagemajik.ai/artanalysis, NAAS will process the files provided and display the results in the "Uploaded Files" section of the page as the processing is complete. If the uploaded figures meet our requirements (or NAAS is able to fix the files to meet our requirements), the figure will be marked as "fixed" above. If NAAS is unable to fix the files, a red "failed" label will appear above. When NAAS has confirmed that the figure files meet our requirements, please download the file via the download option, and include these NAAS processed figure files when submitting your revised manuscript.

**Reproducibility:**

To enhance the reproducibility of your results, we recommend that authors of applicable studies deposit laboratory protocols in protocols.io, where a protocol can be assigned its own identifier (DOI) such that it can be cited independently in the future. Additionally, PLOS ONE offers an option to publish peer-reviewed clinical study protocols. Read more information on sharing protocols at https://plos.org/protocols?utm_medium=editorial-email&utm_source=authorletters&utm_campaign=protocols

Attachment Submitted filename: Review_comments.docx

# Author comment: Author response to Decision Letter 1

**DOI:** 10.1371/journal.pgen.1012300.r002

6 Jul 2026

Attachment Submitted filename: point-by-point response-20260618.docx

# Peer review: Decision Letter 1

**DOI:** 10.1371/journal.pgen.1012300.r003

**Authors:** Angela Hancock, Tomokazu Kawashima

28 Jul 2026

PGENETICS-D-26-00346R1

Multi-organ single-cell transcriptomic atlas identifies QrIAA14 as a candidate negative regulator of adventitious root development in Quercus robur

PLOS Genetics

Dear Dr. Hui,

Thank you for submitting your manuscript to PLOS Genetics. The Reviewers have satisfied with the revision and pointed out several issues to make the story clearer and stronger. Thus, the editorical board requests minor revision without further external review before aacepting the mauscript.

Please submit your revised manuscript within by Aug 27 2026 11:59PM. If you will need more time than this to complete your revisions, please reply to this message or contact the journal office at plosgenetics@plos.org. When you're ready to submit your revision, log on to https://www.editorialmanager.com/pgenetics/ and select the 'Submissions Needing Revision' folder to locate your manuscript file.

Please include the following items when submitting your revised manuscript:

* A letter that responds to each point raised by the editor and reviewer(s). You should upload this letter as a separate file labeled 'Response to Reviewers'. This file does not need to include responses to formatting updates and technical items listed in the 'Journal Requirements' section below.

* A marked-up copy of your manuscript that highlights changes made to the original version. You should upload this as a separate file labeled 'Revised Manuscript with Track Changes'.

* An unmarked version of your revised paper without tracked changes. You should upload this as a separate file labeled 'Manuscript'.

If you would like to make changes to your financial disclosure, competing interests statement, or data availability statement, please make these updates within the submission form at the time of resubmission. Guidelines for resubmitting your figure files are available below the reviewer comments at the end of this letter.

As the corresponding author, your ORCID iD is verified in the submission system and will appear in the published article. PLOS supports the use of ORCID, and we encourage all coauthors to register for an ORCID iD and use it as well. Please encourage your coauthors to verify their ORCID iD within the submission system before final acceptance, as unverified ORCID iDs will not appear in the published article. Only the individual author can complete the verification step; PLOS staff cannot verify ORCID iDs on behalf of authors.

We look forward to receiving your revised manuscript.

Kind regards,

Tomo Kawashima

Academic Editor

PLOS Genetics

Angela Hancock

Section Editor

PLOS Genetics

Aimée Dudley

Editor-in-Chief

PLOS Genetics

Anne Goriely

Editor-in-Chief

PLOS Genetics

**Additional Editor Comments (if provided):**

**Journal Requirements:**

If the reviewer comments include a recommendation to cite specific previously published works, please review and evaluate these publications to determine whether they are relevant and should be cited. There is no requirement to cite these works unless the editor has indicated otherwise.

[Note: HTML markup is below. Please do not edit.]

**Reviewers' comments:**

Reviewer's Responses to Questions

**Comments to the Authors:**

**Please note here if the review is uploaded as an attachment.**

Reviewer #1: The authors have adequately improved their manuscripts and addressed the majority of the points raised in the previous review. I have some minor comments remaining.

1. Methods of phytohormone quantification (from line 647 to 653, and Fig. 5g)

・Please clarify how the samples were collected, specifically, whether they were freeze-dried or simply frozen during sampling. In addition, please describe how the various phytohormones were extracted and purified.

・Which internal standards were used for phytohormone quantification, and in what volume or concentration were they added?

・In lines 651-653 and Fig. 5g: “indole-3-acetic acid (auxin compound)” should be written as “indole-3-acetic acid (IAA)” because "AUX" typically refers to genes or proteins rather than the hormone itself. Similarly, the prefix "trans" in "trans-zeatin" should be italicized and "TZ" should be corrected to "tZ", and "TZR" to "tZR", following standard nomenclature. Additionally, please clarify whether jasmonic acid or jasmonic acid-isoleucine (JA-Ile) was analyzed.

・The vertical axis label in Fig. 5g, "concentration (ng/g)" should be revised to specify the units more precisely, e.g., "concentration (ng/g fresh weight)" or "concentration (ng/g dry weight)," depending on how the samples were processed.

2. Were the cell walls also stained in the sections of in situ hybridization shown in Fig. 6d? If so, please clarify the staining method used.

3. Although red arrowheads have been added in Fig. 6d and described in the manuscript, it is unclear from the figure whether they indicate a specific region or a small signal in R5 stage. Since arrowheads typically indicate a specific point rather than an area, if the intention is to highlight a region, it would be clearer to use a different symbol (e.g., a bracket) instead of arrowheads.

4. In Fig. 6e, the image for "35S:QrIAA14-1.2" is out of focus, so please replace it with a higher-quality image if possible.

5. S5 Table, cell A36 contains the value "10," which appears inconsistent with the surrounding data. Please confirm whether this value is correct. Additionally, the column headers appear to be missing in S5 Table, so please add them for clarity.

Reviewer #2: The authors have made substantial revisions to the manuscript. All previous concerns regarding methodology and data presentation have been adequately addressed. I am satisfied with the quality of the revisions and recommend acceptance.

Reviewer #3: The authors have adequately addressed my previous comments. I have one minor comment regarding the newly added QrIAA–QrARF co-expression analysis.

ARF proteins can function as either transcriptional activators or repressors, and the newly identified QrIAA14-1-associated candidates appear to include members potentially belonging to both classes. This distinction is important for interpreting the proposed QrIAA14-1–ARF regulatory module, because an interaction with an activator-type ARF would have a different mechanistic implication from an association with a repressor-type ARF.

The authors should therefore indicate whether the identified QrARFs, particularly QrARF8-1, are predicted to be activator- or repressor-type ARFs, preferably based on phylogenetic relationships and/or the amino-acid composition of their middle regions. A brief discussion of how this classification relates to the proposed inhibitory effect of QrIAA14-1 on adventitious root elongation would improve the mechanistic interpretation of the newly added analysis. Because these functions have not been experimentally demonstrated in Q. robur, the classification should be presented as putative.

**********

**Have all data underlying the figures and results presented in the manuscript been provided?**

Large-scale datasets should be made available via a public repository as described in the *PLOS Genetics*  data availability policy, and numerical data that underlies graphs or summary statistics should be provided in spreadsheet form as supporting information.

Reviewer #1: Yes

Reviewer #2: Yes

Reviewer #3: Yes

**********

PLOS authors have the option to publish the peer review history of their article (what does this mean?). If published, this will include your full peer review and any attached files.

If you choose “no”, your identity will remain anonymous but your review may still be made public.

**Do you want your identity to be public for this peer review?**  For information about this choice, including consent withdrawal, please see our Privacy Policy.

Reviewer #1: No

Reviewer #2: No

Reviewer #3: No

[NOTE: If reviewer comments were submitted as an attachment file, they will be attached to this email and accessible via the submission site. Please log into your account, locate the manuscript record, and check for the action link "View Attachments". If this link does not appear, there are no attachment files.]

**Figure resubmission:**

While revising your submission, we strongly recommend that you use PLOS’s NAAS tool (https://ngplosjournals.pagemajik.ai/artanalysis) to test your figure files. NAAS can convert your figure files to the TIFF file type and meet basic requirements (such as print size, resolution), or provide you with a report on issues that do not meet our requirements and that NAAS cannot fix.

After uploading your figures to PLOS’s NAAS tool - https://ngplosjournals.pagemajik.ai/artanalysis, NAAS will process the files provided and display the results in the "Uploaded Files" section of the page as the processing is complete. If the uploaded figures meet our requirements (or NAAS is able to fix the files to meet our requirements), the figure will be marked as "fixed" above. If NAAS is unable to fix the files, a red "failed" label will appear above. When NAAS has confirmed that the figure files meet our requirements, please download the file via the download option, and include these NAAS processed figure files when submitting your revised manuscript.

**Reproducibility:**

To enhance the reproducibility of your results, we recommend that authors deposit laboratory protocols in protocols.io, where a protocol can be assigned its own identifier (DOI) such that it can be cited independently in the future. Additionally, PLOS ONE offers an option to publish peer-reviewed clinical study protocols. Read more information on sharing protocols at https://plos.org/protocols?utm_medium=editorial-email&utm_source=authorletters&utm_campaign=protocols

# Author comment: Author response to Decision Letter 2

**DOI:** 10.1371/journal.pgen.1012300.r004

10 Aug 2026

Attachment Submitted filename: Response to Reviewers-20260730.docx

# Editor report: Decision Letter 2

**DOI:** 10.1371/journal.pgen.1012300.r005

**Authors:** Angela Hancock, Tomokazu Kawashima

24 Aug 2026

Dear Dr Hui,

We are pleased to inform you that your manuscript entitled "Multi-organ single-cell transcriptomic atlas identifies QrIAA14 as a candidate negative regulator of adventitious root development in Quercus robur" has been editorially accepted for publication in PLOS Genetics. Congratulations!

Before your submission can be formally accepted and sent to production you will need to complete our formatting changes, which you will receive in a follow up email. Please be aware that it may take several days for you to receive this email; during this time no action is required by you. Please note: the accept date on your published article will reflect the date of this provisional acceptance, but your manuscript will not be scheduled for publication until the required changes have been made.

Once your paper is formally accepted, an uncorrected proof of your manuscript will be published online ahead of the final version, unless you’ve already opted out via the online submission form. If, for any reason, you do not want an earlier version of your manuscript published online or are unsure if you have already indicated as such, please let the journal staff know immediately at plosgenetics@plos.org.

In the meantime, please log into Editorial Manager at https://www.editorialmanager.com/pgenetics/, click the "Update My Information" link at the top of the page, and update your user information to ensure an efficient production and billing process. Note that PLOS requires an ORCID iD for all corresponding authors. Therefore, please ensure that you have an ORCID iD and that it is validated in Editorial Manager. To do this, go to ‘Update my Information’ (in the upper left-hand corner of the main menu), and click on the Fetch/Validate link next to the ORCID field.  This will take you to the ORCID site and allow you to create a new iD or authenticate a pre-existing iD in Editorial Manager.

If you have a press-related query, or would like to know about making your underlying data available (as you will be aware, this is required for publication), please see the end of this email. If your institution or institutions have a press office, please notify them about your upcoming article at this point, to enable them to help maximise its impact. Inform journal staff as soon as possible if you are preparing a press release for your article and need a publication date.

Thank you again for supporting open-access publishing; we are looking forward to publishing your work in PLOS Genetics!

Yours sincerely,

Tomokazu Kawashima

Academic Editor

PLOS Genetics

Angela Hancock

Section Editor

PLOS Genetics

Aimée Dudley

Editor-in-Chief

PLOS Genetics

Anne Goriely

Editor-in-Chief

PLOS Genetics

www.plosgenetics.org

BlueSky: @plos.bsky.social

----------------------------------------------------

Comments from the reviewers (if applicable):

----------------------------------------------------

**Data Deposition**

If you have submitted a Research Article or Front Matter that has associated data that are not suitable for deposition in a subject-specific public repository (such as GenBank or ArrayExpress), one way to make that data available is to deposit it in the Dryad Digital Repository. As you may recall, we ask all authors to agree to make data available; this is one way to achieve that. A full list of recommended repositories can be found on our website.

The following link will take you to the Dryad record for your article, so you won't have to re‐enter its bibliographic information, and can upload your files directly:

http://datadryad.org/submit?journalID=pgenetics&manu=PGENETICS-D-26-00346R2

More information about depositing data in Dryad is available at http://www.datadryad.org/depositing. If you experience any difficulties in submitting your data, please contact help@datadryad.org for support.

Additionally, please be aware that our data availability policy requires that all numerical data underlying display items are included with the submission, and you will need to provide this before we can formally accept your manuscript, if not already present.

----------------------------------------------------

**Press Queries**

If you or your institution will be preparing press materials for this manuscript, or if you need to know your paper's publication date for media purposes, please inform the journal staff as soon as possible so that your submission can be scheduled accordingly. Your manuscript will remain under a strict press embargo until the publication date and time. This means an early version of your manuscript will not be published ahead of your final version. PLOS Genetics may also choose to issue a press release for your article. If there's anything the journal should know or you'd like more information, please get in touch via plosgenetics@plos.org.

# Editor report: Acceptance letter

**DOI:** 10.1371/journal.pgen.1012300.r006

**Authors:** Angela Hancock, Tomokazu Kawashima

PGENETICS-D-26-00346R2

Multi-organ single-cell transcriptomic atlas identifies QrIAA14 as a candidate negative regulator of adventitious root development in Quercus robur

Dear Dr Hui,

We are pleased to inform you that your manuscript entitled "Multi-organ single-cell transcriptomic atlas identifies QrIAA14 as a candidate negative regulator of adventitious root development in Quercus robur" has been formally accepted for publication in PLOS Genetics! Your manuscript is now with our production department and you will be notified of the publication date in due course.

The corresponding author will soon be receiving a typeset proof for review, to ensure errors have not been introduced during production. Please review the PDF proof of your manuscript carefully, as this is the last chance to correct any errors. Please note that major changes, or those which affect the scientific understanding of the work, will likely cause delays to the publication date of your manuscript.

Soon after your final files are uploaded, unless you have opted out or your manuscript is a front-matter piece, the early version of your manuscript will be published online. The date of the early version will be your article's publication date. The final article will be published to the same URL, and all versions of the paper will be accessible to readers.

For Research Articles, you will receive an invoice from PLOS for your publication fee after your manuscript has reached the completed accept phase. If you receive an email requesting payment before acceptance or for any other service, this may be a phishing scheme. Learn how to identify phishing emails and protect your accounts at https://explore.plos.org/phishing.

Thank you again for supporting PLOS Genetics and open-access publishing. We are looking forward to publishing your work!

With kind regards,

Sharmila Kamatchi

PLOS Genetics

On behalf of:

The PLOS Genetics Team

Carlyle House, Carlyle Road, Cambridge CB4 3DN | United Kingdom

plosgenetics@plos.org | +44 (0) 1223-442823

plosgenetics.org | Twitter: @PLOSGenetics
